# Inverse design of frustrated Lewis pairs for direct catalytic CO_2_ hydrogenation: refining and expanding design rules

**DOI:** 10.1039/d5sc09530a

**Published:** 2026-02-05

**Authors:** Shubhajit Das, Ruben Laplaza, Thanapat Worakul, Clémence Corminboeuf

**Affiliations:** a Laboratory for Computational Molecular Design, Institute of Chemical Sciences and Engineering, École Polytechnique Fédérale de Lausanne Lausanne Switzerland clemence.corminboeuf@epfl.ch; b National Centre for Competence in Research–Catalysis (NCCR–Catalysis), École Polytechnique Fédérale de Lausanne Lausanne Switzerland

## Abstract

Frustrated Lewis pairs (FLPs), composed of reactive combinations of Lewis acids (LAs) and bases (LBs) offer a metal-free platform for catalyzing a wide range of chemical transformations. Designing the optimal FLP active site for a particular chemical reaction is a challenging task due to the lack of rigorous principles and countless chemical possibilities. We recently designed principles, which outline the relative disposition (*i.e.*, distance and angle) and chemical composition of the LA and LB centers that maximize activity in B- and N-based FLPs. These criteria were already used to screen 25 000 FLP active sites built on N-containing linkers extracted from the CoRE MOF dataset, but in such an enormous multifunctional catalyst space, inverse design approaches provide a more efficient mean to explore all possible combinations. Here, we use the NaviCatGA genetic algorithm to simultaneously optimize the chemical and geometrical characteristics of intramolecular FLPs while considering synthetic complexity and catalyst quenching constraints. By integrating activity maps and non-linear regression models, our workflow explores a vast chemical space of 1.7 billion FLP candidates built from organic fragments curated from the literature—released as the open-source FragFLP25 dataset—to identify optimal compositions suitable for catalytic CO_2_ hydrogenation. Analyzing the top candidates extracted from various Pareto fronts in the catalyst space, we not only uncover active FLP motifs for hydrogenation that have not been previously reported but also refine and extend the design principles previously established from our high-throughput screening study.

## Abstract 2

Genetic optimization of frustrated Lewis pair catalysts refines design rules and reveals unexplored motifs for CO_2_ hydrogenation.

## Graphical abstract

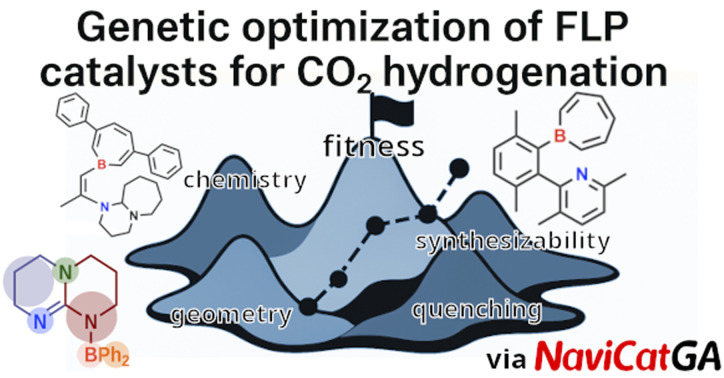

## 1 Introduction

Frustrated Lewis Paris (FLPs) have emerged as promising catalysts for a plethora of metal-free transformations.^1–6^ FLPs feature reactive combinations of Lewis acids (LAs) and Lewis bases (LBs), and their unique reactivity relies on maintaining the LA and LB components in close physical proximity, with dative quenching prevented by steric constraints introduced through substituents and/or geometric constraints imposed by the molecular backbone. FLPs can be realized as standalone molecular catalysts or solid catalysts when the FLP active site is translated into a heterogeneous environment by incorporating the molecular units within rigid scaffolds in porous materials.^7–19^

Recently, some of us proposed a set of intuitive chemical and geometric descriptors to characterize B and N-based FLP active sites, quantitatively mapping their catalytic performance for CO_2_ hydrogenation to formate (CHTF). The chemical and the geometric criteria for optimal activity were found to be: (a) a B–N distance and relative orientation (between the N lone pair and the B empty p orbital) in the range of 2.4–3.2 Å and 70°–140°, respectively^20^ and (b) a specific cumulative acidity and basicity between both sites.^21^ Using these prescribed principles, we conducted a high-throughput virtual screening (HTVS) search of a library of 25 000 FLP active sites^22^ that we built from a top-down curation of the nitrogen-containing organic linkers in the CoRE MOF 2019 database.^23^ While the screening furnished several lead candidates, we noticed the repeated occurrence of a few common structural features among the hits. First, the majority of the active sites featured vicinally-disposed donor–acceptor sites, a finding that is consistent with literature reports of ortho borane-amines being able to reduce CO_2_ (albeit stoichiometrically).^24^ Secondly, the majority of the active sites featured weak to moderately basic sp^2^ N centers as the LB partner. While these trends directly reflect the nature of the nitrogen centers in the parent database, the limited chemical diversity among the hit candidates warrants a more thorough exploration of the chemical space of FLPs. In addition, our screening methodology was executed in a sequential manner, wherein candidates not aligning with our pre-established performance benchmarks were systematically excluded. This approach, however, presents a limitation, as it may overlook catalysts that demonstrate overall high performance yet exhibit suboptimal results when evaluated against a particular criterion.

As an alternative to HTVS,^25–29^ “inverse” design strategies^30–34^ (inspired in part by efforts in drug design^35,36^) start from a set of desired target properties and use an optimization algorithm to generate promising candidates. Recently, generative deep learning approaches^37–52^ have emerged as a promising direction, enabling efficient exploration of large chemical spaces. A wide range of generative deep learning models has been proposed for molecular design applications, such as recurrent neural networks,^51,53,54^ variational autoencoders,^55–57^ and diffusion models.^50,58^ Within this broader landscape, REINVENT^51,54^ a software package for reinforcement-learning-driven molecular generative design and optimization, has become a widely adopted tool and has been successfully applied to molecular design problems spanning drug discovery^59,60^ and functional materials.^61,62^

Nevertheless, genetic algorithms (GA) remain especially well-suited for catalyst discovery, as they explicitly operate on libraries of chemically known and synthesizable fragments, ensuring experimental relevance of candidate molecules. Building on the pioneering efforts of Jensen, Foscato, and coworkers,^63–66^ GA-based inverse design has been further advanced by the groups of Jensen,^67–69^ Balcells,^70,71^ and some of us.^72–75^ Using the NaviCatGA software, we have demonstrated inverse design of molecular materials^75^ as well as catalysts with increased activity, selectivity, or generality.^72–74^ However, the genetic optimization of multifunctional active sites has not been pursued yet.

**Fig. 1:**
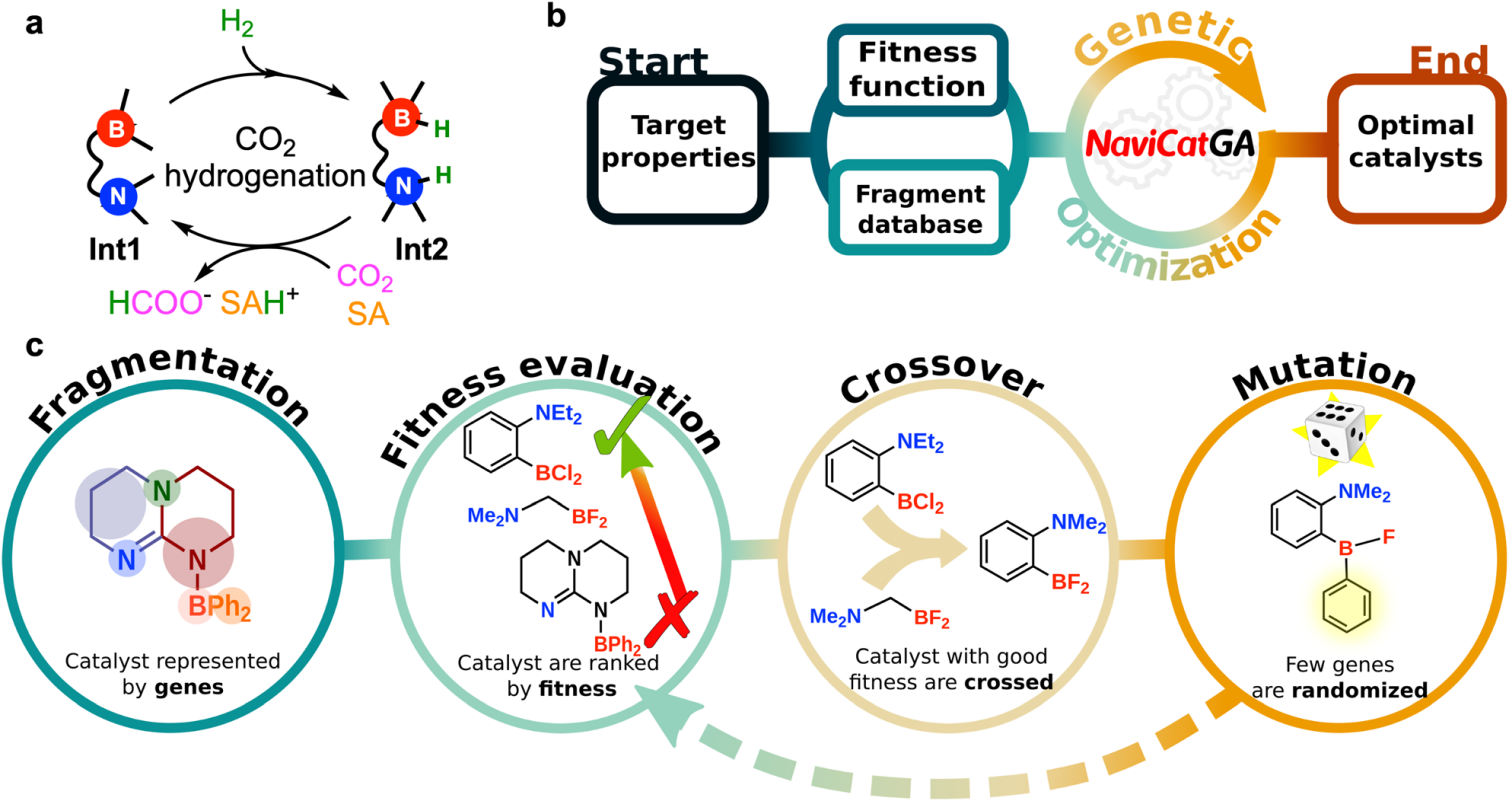
(a) Simplified description of the IFLP-catalyzed direct hydrogenation of CO_2_ to formate. Int1 corresponds to the IFLP catalyst. Int2 corresponds to the product obtained after H_2_ activation, in which the proton and the hydride are attached to the acid and the base centers, respectively. SA represents the sacrificial agent used to drive product release. (b) General workflow for genetic optimization of catalysts (c) Schematic depiction of the steps involved in the genetic optimization of the IFLP catalysts.

In this work, we present an inverse design workflow for the automated discovery of FLP catalysts for direct CO_2_ to formate hydrogenation (Fig. 1a). We focus on intramolecular FLP (IFLP) catalysts where the acid and base units are tethered together in a single molecule. The proposed workflow (Fig. 1b) leverages NaviCatGA^72^ to optimize the chemical and geometrical compositions of IFLPs under experimentally relevant constraints, such as synthetic complexity and resistance to catalyst quenching, while improving performance. This is accomplished by integrating activity maps and non-linear regression models that correlate structure to properties. Our workflow explores a vast chemical space of 1.7 billion IFLP candidates to uncover optimal compositions suitable for catalyzing CO_2_ reduction. Analysis of the leading candidates obtained from the Pareto fronts across the catalyst space reveals previously unreported, active FLP motifs for CHTF, while simultaneously refining and broadening the design principles identified in our earlier HTVS study.

## 2 Methods

In the genetic optimization of FLP catalysts (Fig. 1c), a candidate (“chromosome”) is composed of individual fragments (“genes”). A set of randomly generated catalysts (“population”) are computationally evaluated based on their suitability for CHTF (their “fitness”) using a highly tailored, scalarized fitness function (*vide infra*). The worst-performing catalysts are discarded, while the best-performing ones are recombined with each other (“crossover”), and randomly mutated into new ones to form the next generation in the evolution. The process continues until the performance does not further improve or a fixed number of generations has been reached.

The methods section is organized as follows: Section 2.1 describes various components involved in the genetic optimization workflow (Fig. 1b and c) that search for IFLP compositions that yield the optimal values of several desired target properties, such as the chemical and geometric composition of the catalysts, synthesizability, and resistance towards known catalyst deactivation pathways. These include defining a chemical space for IFLP catalysts, multiple fitness functions that map catalyst composition and geometry to performance, and definitions of the multiple objectives for genetic optimization (Sections 2.2 and 2.3). All codes related to the optimization workflow, including the machine learning models and the data from the optimization runs, are made publicly available in https://github.com/lcmd-epfl/ga_flp.

### 2.1 Creation of the chemical space for IFLPs—the FragFLP25 database

Each IFLP catalyst (chromosome) is composed of eight genes. The genes are associated with the different fragments of an IFLP catalyst. Specifically, there are two LA substituent genes bound to the boron center; two LB substituent genes bound to the nitrogen center; one backbone gene defining the covalent tether between the acid and base moieties, and three optional backbone-substituent genes that functionalize predefined positions along the backbone (see Scheme S1 for a visual depiction). All genes are represented as SMILES fragments with explicit attachment points, ensuring chemically valid connectivity during assembly.

Depending on the topology encoded by the backbone gene, namely, whether the boron and/or nitrogen atoms are exocyclic or endocyclic, not all genes are expressed. In such cases, genes corresponding to chemically inaccessible positions are automatically silenced and excluded from both structure construction and fitness evaluation. This conditional gene expression allows a single chromosome representation to uniformly encode noncyclic FLPs as well as FLPs featuring endocyclic donor–acceptor systems without introducing chemically invalid structures.

These fragments were curated from the prior FLP literature and span a wide range of steric and electronic environments. We examined several publications relevant to FLP chemistry in depth and isolated common substituents found in the LA and LB centers, as well as relevant organic backbones.^1,3,5,6,76–78^ These collections were complemented by additional chemical motifs frequently observed in organo(metallic)catalysis. The final combinatorial pool is built from 71 acid substituents, 40 LB substituents, 108 backbones, and 2 backbone substituents, leading to a chemical space of over 1.7 billion possible IFLPs. In comparison, we scanned the FORMED database, which contains a massively diverse set of 117 K synthesized organic molecules mined from the CSD,^79^ and found only 26 different backbones among 332 IFLP structures in total, a fraction of what the FragFLP25 database contains. This motivated us to build the combinatorial space by directly incorporating literature precedents.

Assembly of the full IFLP structure is performed by a deterministic assembler function that concatenates the chromosomes into SMILES of IFLP. The resulting SMILES string is then converted into a 3D structure for fitness evaluation. The robustness of the assembler function is exemplified by its ability to construct various classes of FLPs, including exocyclic and endocyclic donor–acceptor systems (*vide infra*). All organic fragments (represented as SMILES strings) used to build the FLPs are available at https://github.com/lcmd-epfl/ga_flp as the FragFLP25 database.

### 2.2 Evaluation of fitness of an IFLP catalyst

After defining the catalyst space through fragmentation, we must associate one (or several) appropriate fitness score(s) to drive the genetic optimization towards optimal candidates. In the present case, the fitness function must map the structure of catalysts to their CHTF performance, which requires satisfying several distinct objectives. These objectives are achieving optimal (i) chemical and (ii) geometrical composition for CHTF, while ensuring (iii) synthetic accessibility and (iv) resistance to dimeric quenching. Fitness evaluation proceeds by first generating a 3D structure of the FLP catalyst bound to a hydride and a proton (*i.e.*, Int2 which captures the geometric response of the FLP environment to the inclusion of the H_2_ molecule)^20^ from the SMILES string contained in the chromosome using distance geometry methods as implemented in RDKit,^80^ and optimizing Int2 at the GFN2-xTB level.^81^ Electronic, geometric, and steric descriptors are then extracted from the optimized structure and used in linear and non-linear predictive models to compute the final fitness score. In what follows, we describe each objective in detail and how all four objectives are incorporated into the fitness score used by the GA.

#### 2.2.1 Chemical fitness

**Fig. 2:**
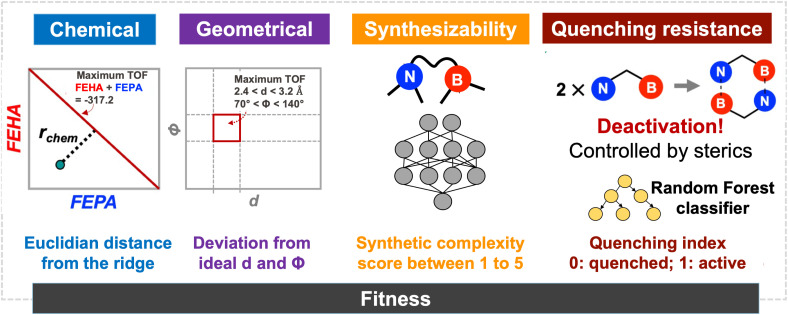
Evaluation of fitness scores considering the four main aspects – chemistry, geometry, synthetic complexity, and catalyst deactivation pathways – that control the catalytic activity of IFLPs.

In a previous work, we established a relationship between the chemical composition of Lewis pairs and their CHTF activity based on the acidity and basicity of the Lewis centers, as estimated from two chemical descriptors: the free energy of hydride attachment (FEHA) and the free energy of proton attachment (FEPA) of the corresponding acid and base centers, respectively,^21^ as computed at the PBE0-D3(BJ)/def2-TZVP level.^82–85^ To ensure high catalytic activity, the acidity and basicity of the Lewis components require complementarity. In other words, the cumulative acid–base strength of the pair dictates CHTF activity, regardless of the individual strength of the components. The relevance of our model was confirmed by the experimental demonstration of the first reported catalytic turnovers in FLP-catalyzed hydrogenation of CO_2_.^21^ The chemical fitness of candidates is evaluated by exploiting this previously established computational framework. The balance between the chemical descriptors and the theoretically derived turnover frequency (TOF)^86^ for the CHTF cycle is given by the criterion FEPA + FEHA = −317.2 kcal mol^−1^, as shown schematically in Fig. 2 and described in detail elsewhere.^22^ The red line across the map indicates the desired complementarity between acidity and basicity, leading to maximum TOF for CHTF. The chemical fitness of a given candidate *i* is estimated by measuring the Euclidean distance *r*_chem_ from this line, as1
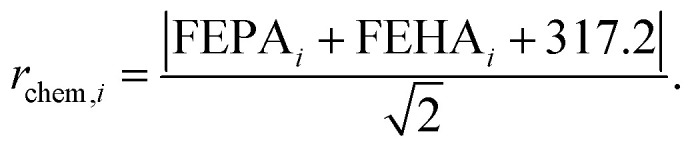


DFT-accuracy FEPA and FEHA are needed, but the DFT-level estimation of chemical fitness is expensive and not suitable as such for implementation into a GA pipeline, where fitness evaluation must be as fast as possible. The computational cost is reduced using a linear correction to GFN2-xTB to estimate the desired PBE0-D3(BJ)/def2-TZVP energies. Fig. S1 shows the correlation between the FEPA/FEHA values at the GFN2-xTB and PBE0-D3(BJ)/def2-TZVP levels. The excellent correlation diagnostic between these two quantities (*R*^2^ = 0.90, 0.94) allows for a quick estimation of the chemical descriptors (see Fig. S1).

#### 2.2.2 Evaluation of geometrical fitness

The relationship between the active site geometry of the IFLP and CHTF activity has been investigated in depth.^20^ It was found that activity can be expressed as a function of two physically intuitive geometrical descriptors, namely the distance (*d*) and orientation (angle, *Φ*) of the donor–acceptor units assumed during the catalytic cycle.^20^ While *d* is trivially calculated from the distance between the donor and acceptor centers, *Φ* is estimated from the angle between their open coordination sites (*i.e.*, the lone pair of the base and the empty p orbital of the acid) for substrate binding. The geometric criteria for high CHTF activity stipulate that the LPASs featuring B–N distances between 2.4 and 3.2 Å and relative orientations between 70 and 140° are substantially more reactive than the others.^20^ The descriptors are extracted from intermediate Int2 of the CHTF catalytic cycle to capture the effect of the reaction environment, *i.e.*, the inclusion of the H_2_ molecule (see Fig. 1a).

The geometrical fitness of a candidate FLP *i* is assessed by comparing its *d* and *Φ* to their respective target values using a normalized Gaussian function:2
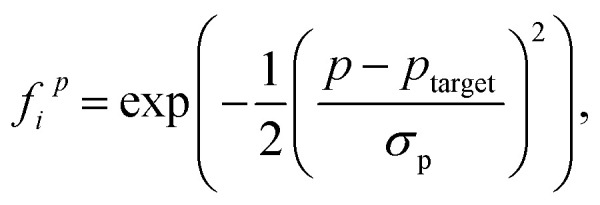
where *p* ∈ *d*, *Φ* is the geometric attribute of interest for candidate *i*, *p*_target_ is the desired target value. In all optimization trials, the width parameters were fixed to *σ*_*d*_ = 0.60 and *σ*_*Φ*_ = 0.75 for *d* and *Φ*, respectively.

The overall geometrical fitness score *G*_*i*_ is then computed as a weighted sum of the individual distance and angle scores:

3 *G*_*i*_ = *C*_1_*f*_*i*_^*d*^ + *C*_2_*f*_*i*_^*Φ*^,

where *C*_1_ and *C*_2_ are weighting coefficients reflecting the relative importance of distance and angle terms. In this work, both are set to 0.5 to assign equal weight.

#### 2.2.3 Synthetic complexity

To avoid generating molecules that are experimentally irrelevant (*e.g.*, with unconventional or exceedingly complex topologies) and favor the simplest catalyst if all other factors are equal, a synthetic complexity score (SCS) is included in the fitness function. For any given candidate, their SCS is predicted using the neural network model developed by Coley *et al.*^87^ This model assigns a score between 1 and 5, with higher values suggesting molecules that are more complex. The SCS must be minimized in the optimization process. It must be noted that the model was developed under the premise that molecules that are synthesized from other molecules must have a higher score than those, which means that a higher score is associated to higher molecular complexity – and, to a degree, molecular size –, but not necessarily nonphysical or unstable molecules.^79^

#### 2.2.4 Resistance to quenching

The CHTF activity for IFLPs is hindered by intramolecular quenching of the LA and LB centers or intermolecular quenching involving two or more molecules, which constitutes an off-cycle deactivation route for the catalytic cycle. Such classical adduct formation is controlled by the steric availability of the centers, both within the molecule and between different IFLP molecules.

To determine whether classical adduct formation between a pair of acid and base centers is possible or not, we developed a random-forest classifier based on the steric bulk around the Lewis centers. We curated a dataset of 647 LA and LB combinations using diverse substituents (Fig. S7 and S8). Each pair was labeled as frustrated or quenched on the basis of a Boltzmann weighted LA–LB distance (BWDistance) that is extracted from the geometries and energies of several geometry optimization runs started with different initial distances for the LA and LB pair. We observed a strongly bimodal distribution where most pairs feature BWDistance clearly below or above the 2.0 Å mark, which we therefore used as our decision boundary (see Fig. S10). While this criteria, based on BWDistance, does not account for the kinetic aspects of frustration, it captures thermodynamic preference: pairs where the formation of a strongly favorable dative interaction are unmistakably driven to BWDistance of about 1.6 Å, whereas sterically encumbered combinations lead to significantly larger distances. Only about 3% of the LA/LB combinations examined had an ambiguous character that could be decisively influenced by kinetics or other effects.

As input features for the classification model, each Lewis center is described by a set of four descriptors representing the buried volumes^88–90^ in increasingly large radii around the atomic position (2.0, 2.5, 3.0, and 3.5 Å). The classification is binary: for a given LA and LB pair, we predict whether a classical adduct could be prevented (quenching index *Q* = 1) or not (*Q* = 0). In 20-fold cross-validation studies, we found the overall accuracy to be over 95% accurate in correctly identifying combinations that could lead to quenching due to lack of frustration (see Fig. S11). In turn, this implies that the inclusion of the dimer quenching classifier in our fitness function is unlikely to (falsely) accept problematic candidates. See SI for more details on the training, cross-validation, and limitations of the random forest classifier.

### 2.3 Multi-objective optimization and scalarization

Here, we describe the implementation of the multiobjective GA-based optimization of the IFLPs for CHTF. To find the optimal catalyst, the geometric and chemical suitability is maximized while minimizing the SCS and ensuring that the quenching model predicts no intermolecular classical adduct formation. In mathematical terms, the optimization aims at simultaneously maximizing two objectives, minimizing a third, and complying with a classifier. Such a multi-objective setting is challenging, even more so if trade-offs are anticipated between the different objectives. In our case, some objectives may be compatible, such as chemical fitness and high complexity, as there is no intrinsic reason to assume that the tuning of acidity and basicity of the LA and LB centers will increase the synthetic complexity. However, other combinations, such as complexity and quenching, lead to trade-offs, as illustrated in Fig. S3, because larger, more elaborate molecules may improve resistance to quenching while raising the SCS, for instance. Among all four objectives, multiple convoluted trade-offs will exist, leading to several Pareto fronts in which some objective(s) must be degraded in order to improve other(s). Since which point of the front that will be found by the GA depends on the relative preference allocated to each of the different objectives, we explore different priorities in the different GA runs using the hierarchical scalarizer Chimera^91^ (*vide infra*).

The multiobjective optimization is driven on the basis of three scores as defined below:

• Chemistry score (CS): defined as the inverse of the Euclidean distance from the maximal TOF line multiplied by the quenching index, 1/*r*_chem_ × *Q*.

• Geometry score (GS): defined as the distance–angle score multiplied by the quenching index, *G* × *Q*.

• Synthetic complexity score (SCS) as estimated from Section 2.2.3.

The optimization attempts to maximize CS and GS while minimizing SCS simultaneously. In simple terms, we optimize objectives hierarchically following a fixed order in each run. Within that priority order, the scalarizer examines the candidates where the first objective is within a given relative tolerance of the best case seen so far. In those cases, it considers the second objective with its own independent relative tolerance, and so on for the third. Afterwards, from the cases where all values of CS, GS, and SCS are within their respective tolerances, it selects the case that better satisfies all requirements. We refer the reader to the original publication of the hierarchical scalarizer Chimera^91^ for further details, and see Table S1 for details on scalarization settings for each individual reported.

## 3 Results and discussion

**Fig. 3:**
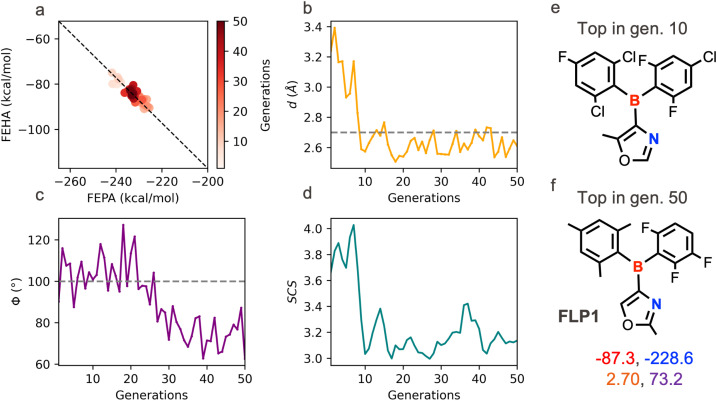
Evolution of the chemical descriptors, FEPA and FEHA (a), geometric descriptors, *d* and *Φ* (b and c), and SCS (d) for a multiobjective optimization run by an objective hierarchy CS, GS followed by SCS. The average values of the properties over the population are shown. The black dotted line in the chemical descriptor space in Fig. 4a depicts the desired complementarity between FEPA and FEHA to achieve maximum TOF. The grey dashed lines in b and c indicate the target *d* and *Φ* values, respectively, used in the optimization. The top candidate extracted after the 10th generation is shown in (e). The top candidate extracted after the 50th generations, along with four descriptor values (*d*: orange, *Φ*: violet, FEHA: red, FEPA: blue) is shown in (f).

The first set of multiobjective optimization runs are performed with a population size of 20 randomized individuals and a mutation rate of 0.1 for 50 generations. This setting corresponds to the evaluation of 1000 IFLPs at most, which is a minuscule fraction of the combinatorial search space of 1.7 B candidates. The fitness scores are scalarized using a 25%, 10%, and 25% degradation threshold for CS, GS, and SCS, respectively, which prioritizes the chemistry objective but keeps the geometry criteria as a close second. The target values for the geometric descriptors *d* and *Φ* are kept at 2.7 Å and 100°, respectively. Fig. 3 illustrates the evolution of the relevant descriptors that track the progress of the optimization. As the optimization proceeds, the population gradually migrates diagonally downwards and then upwards over the 50 generations, remaining reasonably close to the line corresponding to the maximum TOF (Fig. 3a). While the target *d* value is nearly reached within 50 generations, the optimization falls short of achieving the target *Φ*; however, it remains within the prescribed range of high activity based on the geometric descriptors (Fig. 3b and c).

Simultaneously, the SCS decreases steadily, implying that the GA finds simpler molecules while optimizing both chemistry and geometry (see Fig. 3d). A multiobjective trade-off emerges at around generation 10, coinciding with the discovery of an oxazole backbone, which brings the geometry of the active site close to the target values while producing a sharp drop in SCS. The subsequent generations focus on fine-tuning both the electronic and steric components of the active site by exploring suitable substituents on the borane and oxazole motif. The top candidate, FLP1, after 50 generations, features an oxazole core positioning the boron and the nitrogen centers in a geminal fashion. The weak basicity of the pyrrolic nitrogen (from the oxazole) is delicately balanced by an appropriately strong LA unit featuring an aryl-borane with a 2,4,6-trimethylphenyl (*i.e.* mesityl), a 2,3,6-trifluorophenyl as the substituents- both of which have been previously reported in FLP literature.^3^ The computed FEPA and FEHA values for this FLP are −228.6 and −87.3 kcal mol^−1^, respectively, leading to a cumulative acid–base strength of −315.9 kcal mol^−1^, which is very close to the previously identified optimum (−317.2 kcal mol^−1^) associated with high activity. The geometric descriptors for FLP1 are estimated to be 2.7 Å and 73.1°. The computed energy span,^86^ which is an estimate of the most difficult energetic step in the catalytic cycle, for FLP1 is 28.9 kcal mol^−1^, which is consistent with the estimated optimal value of the chemical and geometrical descriptors (see Fig. S4).

**Fig. 4:**
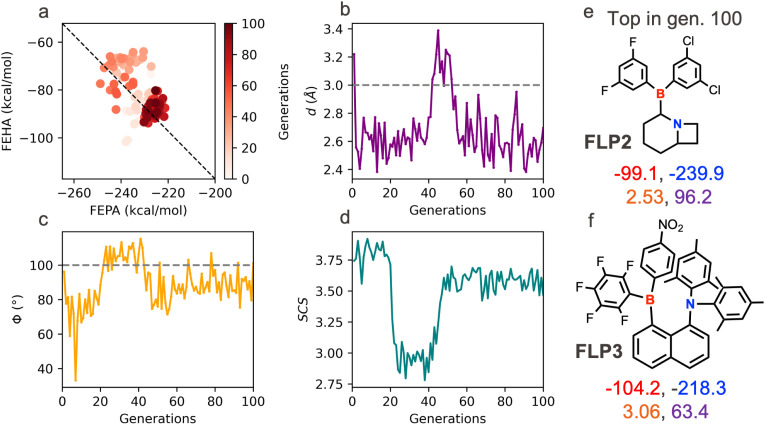
Evolution of the chemical descriptors, FEPA and FEHA (a), geometric descriptors, *d* and *Φ* (b and c), and SCS (d) for a multiobjective optimization run by an inverted objective hierarchy GS, CS followed by SCS. The average values of the properties over the population are shown. The black dotted line in the chemical descriptor space in (a) depicts the desired complementarity between FEPA and FEHA to achieve maximum TOF. The grey dashed lines in (b) and (c) indicate the target *d* and *Φ* values, respectively, used in the optimization. The top candidate extracted after 100 generations is shown on the right (e). Top candidate obtained in another optimization run (for the details see Fig. S2) with the same settings but using different target values is shown on the bottom (e). The corresponding four descriptor values (*d*: orange, *Φ*: violet, FEHA: red, FEPA: blue) for the FLPs shown in (e and f) are mentioned at the bottom.

For the second set of runs, we invert the objective ordering to prioritize GS over CS instead. Fig. 4 and S5 show the results of two such runs. For the run shown in Fig. 4, the population explores a more diverse region in the chemical space compared to the previous run in order to prioritize the geometric environment of the active site. At the end of 100 generations, both runs achieve the desired target in the *Φ* value, while it falls short of the distance target, although remaining within the prescribed range of high activity. As expected from these optimization settings, the two top FLP candidates found at the end of those optimizations feature a heavily constrained or sterically congested FLP active site that enables the precise geometric environments to perform the CHTF. The candidate FLP2 features a mixed halide-substituted aryl borane unit as the LA hosted on a 1/7-azabicyclo[4.2.0]octane scaffold. The constrained nitrogen site at the junction of the six- and 4-membered rings and the relative geminal positioning with respect to the boron center furnish *d* and *Φ* values of 2.53 Å and 96.2°, respectively, which is highly appropriate for CHTF. The top candidate from the other optimization run, FLP3, features an FLP active site where an aryl borane LA and a bulky dimesityl-bearing amine LB are placed on the bay positions of a naphthalene motif. The large substituents present on both the B and N centers create a sterically congested yet appropriate geometric environment for CHTF (*d* and *Φ* values of 3.06 Å and 63.3°, respectively). Since CS is the secondary target, for both FLP2 and FLP3, the cumulative acid–base strengths are suboptimal compared to FLP1. As a result, despite having appropriate active site geometry, the overall energy span of the CHTF profiles are greater than 35 kcal mol^−1^ (see Fig. S4), which is significantly higher than that of FLP1.

To examine the effect of considering the quenching index in the fitness function, we have performed GA runs only based on 1/*r*_chem_, *G*, and SCS as the objectives to satisfy the chemistry, geometry, and synthetic complexity-based criteria, respectively. Our results reveal that, without the quenching constraint, often less sterically encumbered donor or acceptor centers are found as the top candidates. Fig. S6a depicts one such representative run where the top candidate features a combination of a B center bearing a Cl and a vinyl ligand with pyridine N site in a geminal position. The progress of the optimization indicates difficulties in achieving the target *d* and *Φ* values while the SCS is relatively easily minimized to a comparatively lower value (with respect to when *Q* is included in the fitness function) in the absence of the trade-off with the *Q*. A somewhat similar effect is observed with the quenching score included in the fitness functions but thorough optimization by prioritizing minimization the SCS as the first objective followed by maximization of the chemistry and geometry scores, respectively. In these runs, structurally less complex Lewis pairs are prioritized, and often these are sterically less constrained with a possibility of intermolecular quenching in the solution (Fig. S6).

**Fig. 5:**
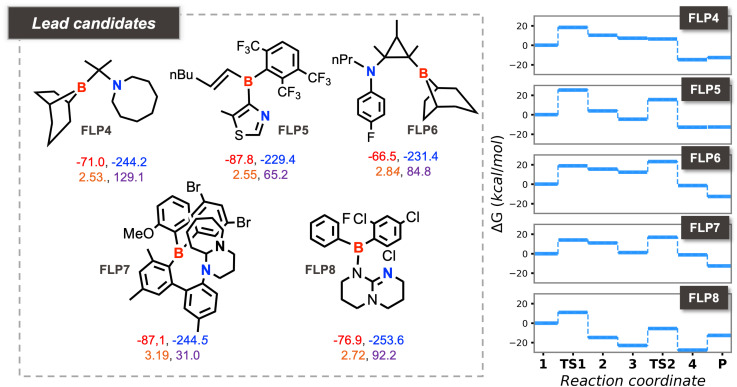
Top candidates obtained from various optimization runs (for the details of the settings, see Table S1) along with the corresponding four descriptor values (*d*: orange, *Φ*: violet, FEHA: red, FEPA: blue). The right panels show the explicitly computed CHTF profiles for these lead candidates.

Compared to our earlier HTVS study, which applied sequential filters based first on geometrical and then on chemical appropriateness for high CHTF activity, the present work explores the chemical space by simultaneously optimizing both factors. Fig. 5 highlights the top candidates identified from several multiobjective optimization runs, each probing distinct regions of the chemical landscape. From the lead candidates previously identified through HTVS,^22^ we derived pragmatic, experimentally relevant design rules for highly active Lewis pair sites. However, the accessible chemical space was inherently limited by two factors: (1) the restricted chemical and structural diversity of nitrogen sites within the experimental MOF database used to curate organic fragments and preselected borane units, and (2) the sequential use of geometrical and chemical descriptors, which favored candidates performing well overall, but candidates that perform suboptimally with respect to individual criteria were all filtered out. By contrast, the present multiobjective approach removes these constraints and concurrently optimizes all relevant descriptors, thereby uncovering systems that eluded the earlier HTVS. Analysis of these discovered structures enables us to refine and expand the existing design rules, providing a more complete picture of the structure–performance relationships for Lewis pair catalysis in CHTF.

**Scheme 1:**
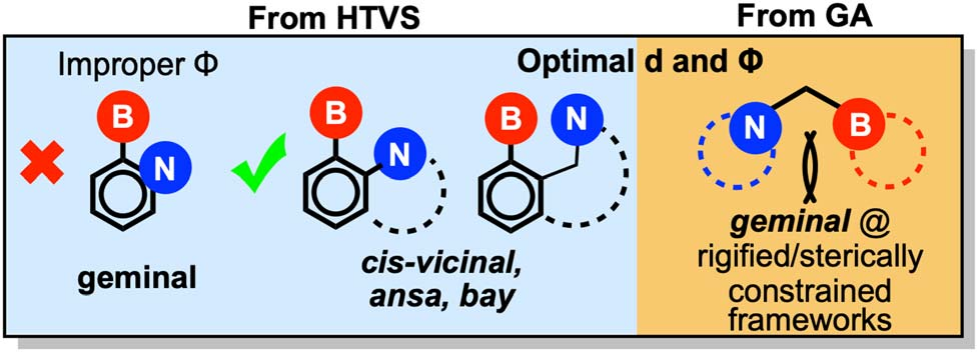
Modified geometric design rules obtained from HTVS (left, light blue portion) *vs.* GA (right, yellow portion).

The geometric design rules derived from our previous HTVS study indicated that *cis*-vicinal and ansa/bay arrangements—where the boron and nitrogen centers are separated by two or three atoms—offer the most favorable configurations for CHTF (Scheme 1). Geminal FLPs, in which the two centers are separated by a single atom, were instead found to be ineffective. Although these systems meet the optimal B–N distance criterion (2.5–2.7 Å), their relative orientations produce small *Φ* angles that hinder H_2_ activation and product release, both previously linked to poor catalytic performance. Consequently, no geminal FLPs appeared among the HTVS lead candidates, despite their abundance in the initial screening pool.

In contrast, the present multiobjective optimization uncovered several promising geminal FLPs, including FLP1, FLP4, and FLP5. As shown in Fig. 1 and 5, some of these systems exhibit unexpectedly favorable *Φ* values (70–140°) despite their geminal geometry. In most cases, rigidity within the local framework—arising from endocyclic incorporation of the donor/acceptor centers or sterically demanding substituents—stabilizes the favorable orientation. For example, FLP4 displays a remarkably large *Φ* of 129°, enforced by a boron center on the bridgehead of a bicyclic scaffold and a nitrogen center within an azacyclooctane ring. Similarly, steric congestion around the boron center along with heterocyclic nitrogen LB sites in FLP1 and FLP5 yields a *Φ* of 73 and 65°, respectively. These geminal FLPs exhibit cumulative acid–base strengths reasonably close to the optimal range (−315.9, −315.2, and −317.2 kcal mol^−1^, for FLP1, FLP4, and FLP5 respectively). In line with the estimated descriptor values, FLP4 displays the most favorable CHTF profile, exhibiting the lowest energy span (20.5 kcal mol^−1^) of the three geminal FLPs.

The GA also identified a distinct candidate, FLP7, featuring acid–base centers separated by four carbon atoms. These motifs found by the GA substantially enrich the structural diversity of viable FLPs for CHTF, revealing geometric solutions inaccessible to sequential screening approaches.

**Fig. 6:**
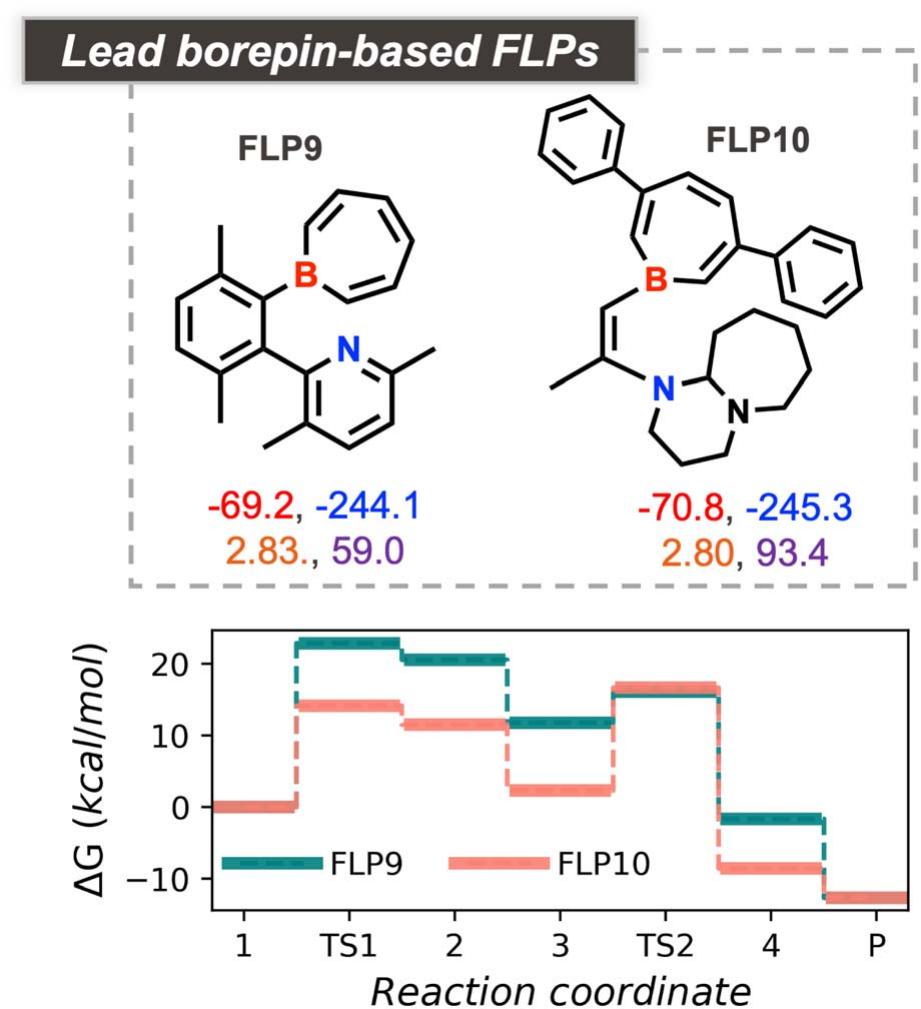
Top borepin-based FLP candidates, along with the corresponding four descriptor values (*d*: orange, *Φ*: violet, FEHA: red, FEPA: blue), obtained from the multiobjective optimization runs. The bottom panel shows the explicitly computed CHTF profiles.

Beyond refining established geometric design rules, the current workflow also uncovers chemically exotic FLPs that, to the best of our knowledge, have not been reported experimentally. One particularly intriguing candidate, FLP9, features a borepin-based Lewis acid paired with a pyridinic nitrogen Lewis base situated in an ansa position (see Fig. 6). Both its cumulative acid–base strength (FEPA + FEHA = −313.3 kcal mol^−1^) and geometric descriptors (*d* = 2.83 Å, *Φ* = 59.0°) closely approach the previously identified optimal values. The computed free-energy profile yields an energy span of 22.8 kcal mol^−1^, with 1 and TS1 identified as the turnover-determining intermediate (TDI) and transition state (TDTS), respectively.

A related borepin-containing FLP, FLP10, emerged in another optimization run targeting a shorter B–N separation of 2.7 Å, corresponding to a vicinal arrangement of the acid and base centers (see Fig. 6). In this system, the Lewis acid site features a substituted borepin, while the Lewis base is replaced by a tertiary amine derived from a 1,8-diazabicyclo[5.4.0]undecane motif. The cumulative acid–base strength (−316.1 kcal mol^−1^) is even closer to the optimal range, and the rigid ethylene bridge enforces a *d* value of 2.79 Å with an improved *Φ* of 93°. The computed CHTF free-energy profile confirms enhanced reactivity, exhibiting an energy span of 16.6 kcal mol^−1^—lower by 6.2 kcal mol^−1^ compared to the borepin–pyridine analogue. We find a mean SCS of 3.50 ± 0.55 (see Table S2 for individual SCS) for the set of top candidates FLP1-10, which indicates that the generated candidates are complex but reasonable, particularly given that their constituent fragments were curated from the literature. As a comparison, it is worth mentioning that the mean SCS of the FORMED database was estimated to be 2.8 ± 0.8 even though these molecules without exception have been synthesized and crystallized.^79^

These results highlight the capability of the present workflow to navigate beyond conventional design spaces and identify unconventional yet catalytically viable FLPs, underscoring the power of multiobjective genetic optimization in discovering unconventional chemical motifs for CHTF. Owing to the simplicity of the GA and the modular nature of our optimization workflow, the framework can be readily adapted to discover FLP catalysts for a broad range of industrially relevant transformations.

## 4 Conclusions

In conclusion, we presented an inverse design workflow for the automated discovery of FLP catalysts for the direct hydrogenation of CO_2_ to formate. The proposed workflow is based on a genetic algorithm that optimizes the chemical and geometrical compositions of the catalysts under experimentally relevant constraints, such as synthetic complexity and catalyst quenching pathways, towards improving the activity. This is achieved by combining activity maps with non-linear regression models to relate geometrical and chemical features to catalytic performance. The workflow explores a vast chemical space of 1.7 billion FLP candidates to uncover optimal compositions suitable for catalyzing this transformation. By performing various evolutionary experiments, we precisely identify the structural changes that are advantageous to improving their performance and their influence on the overall activity. The analysis of the top candidates extracted from the various Pareto fronts in the catalyst space not only uncovers uncommon active FLP motifs for CHTF but also refines and extends the design principles previously established from our HTVS study. Notably, the algorithm discovers several geminal FLPs—previously dismissed as unsuitable due to unfavorable acid–base orientations—that achieve optimal active site geometries through rigidified or sterically constrained frameworks. In addition, we further uncover a previously unexplored Lewis acid motif in FLP chemistry—the borepin-based unit, which, when paired with aliphatic or heterocyclic amines, emerges as one of the most promising candidates for CHTF. The range of SCS values observed suggests that top FLP candidates are complex yet reasonable molecules, and all candidates are composed of known building blocks of the FragFLP25 database by design. The fragment-based, bottom-up construction strategy generates less exotic structures than fully generative approaches, while ensuring candidates are composed of motifs with established synthetic precedents.

The chemical and geometric descriptors are expected to be directly transferable across a broad range of FLP-catalyzed hydrogenation reactions, as many key design criteria-such as the acidity and basicity of the components and their spatial arrangement-are shared among these systems. While the associated fitness functions may require quantitative reparameterization for a specific catalytic transformation, the underlying descriptors remain generally applicable. Another component of the fitness function, the quenching score, *Q*, typically, represents a necessary condition for effective FLP catalysis, as it prevents off-cycle catalyst deactivation. Consequently, the binary classifier developed in this work is transferable and integrable into any suitable molecular discovery workflow. In general, the genetic algorithm-driven inverse design of catalysts through the concurrent optimization of multiple objectives, coupled to data-driven predictive models based on different architectures and underlying data, is a powerful and pragmatic approach for fragment-based catalyst design and will enable future large-scale computer-aided discovery efforts in catalysis.

## Author contributions

Conceptualization, S. D., R. L. and C. C.; methodology, S. D., R. L., and T. W.; investigation, S. D, R. L., and T. W.; software, S. D., R. L. and T. W.; formal analysis S. D and R. L.; writing – original draft, S. D. and R. L.; writing – review and editing, S. D., R. L., T. W. and C. C; supervision, and funding acquisition, C. C.

## Conflicts of interest

There are no conflicts to declare.

## Supplementary Material

SC-017-D5SC09530A-s001

SC-017-D5SC09530A-s002

## Data Availability

The code used for training the dimer quenching classifier and performing the genetic optimization runs is available on GitHub at https://github.com/lcmd-epfl/ga_flp. The FragFLP25 database and the dataset used to train the classifier can be found at https://github.com/lcmd-epfl/ga_flp/tree/main/data. Supplementary information (SI) is available. See DOI: https://doi.org/10.1039/d5sc09530a.
