# Supplementary figures and images for: Inverse design of frustrated Lewis pairs for direct catalytic CO2 hydrogenation: refining and expanding design rules
Source: Chem Sci. 2026 Feb 5;17(14):7071–81. doi: 10.1039/d5sc09530a (PMC12903909; doi:10.1039/d5sc09530a)

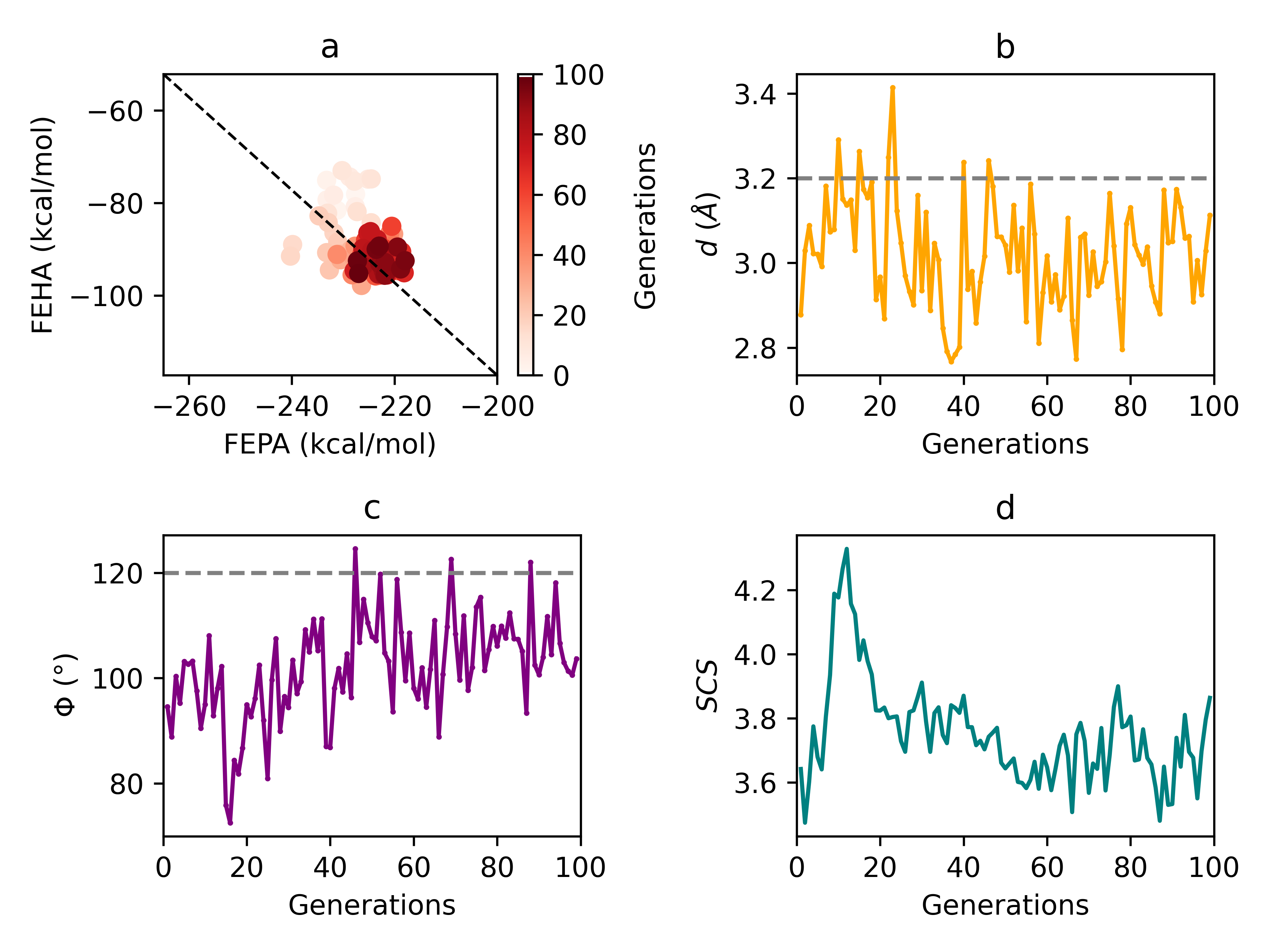

Supplement: SC-017-D5SC09530A-s001 [file SC-017-D5SC09530A-s001.zip › figures_si/62_gcs_2_26.png]

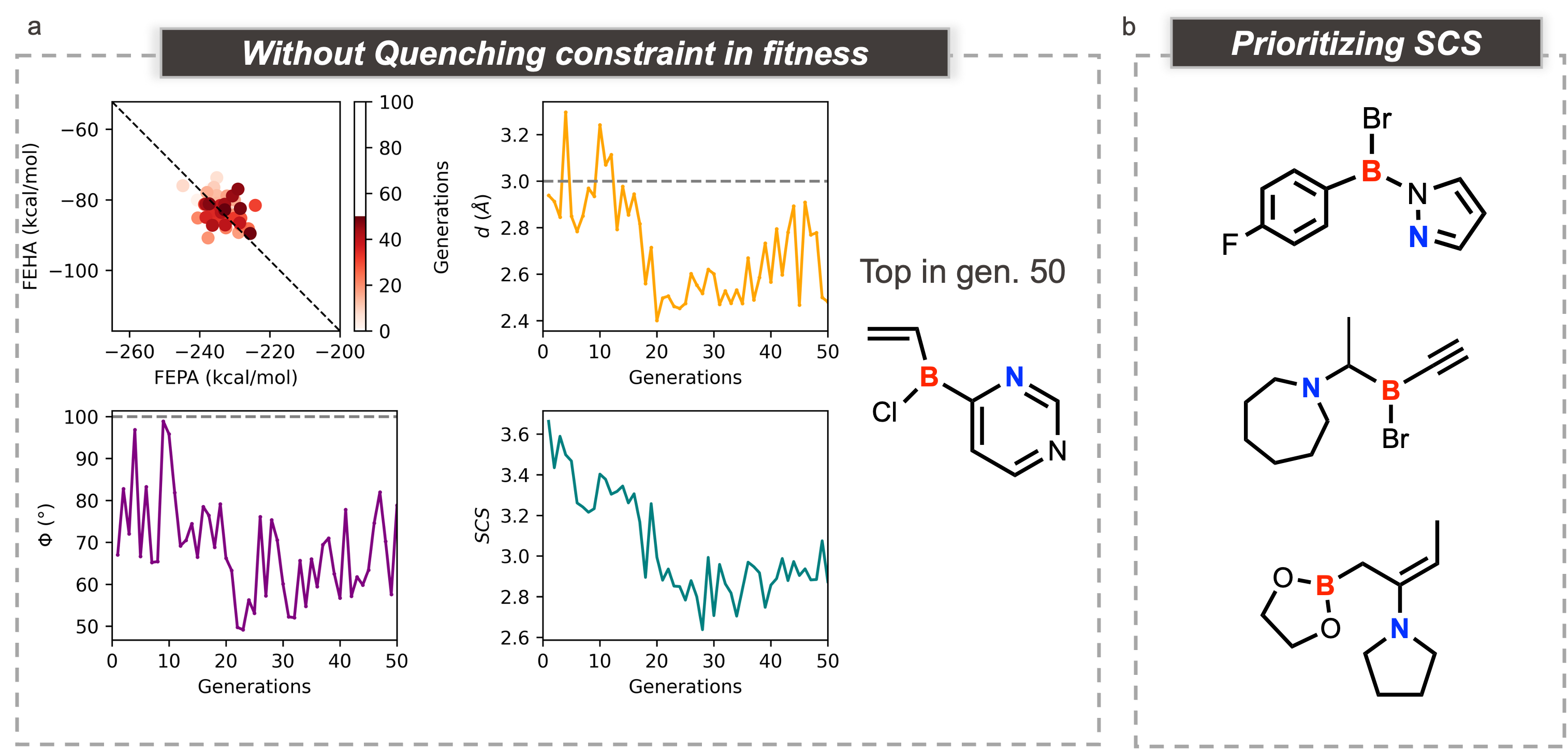

Supplement: SC-017-D5SC09530A-s001 [file SC-017-D5SC09530A-s001.zip › figures_si/62_wofr_7.png]

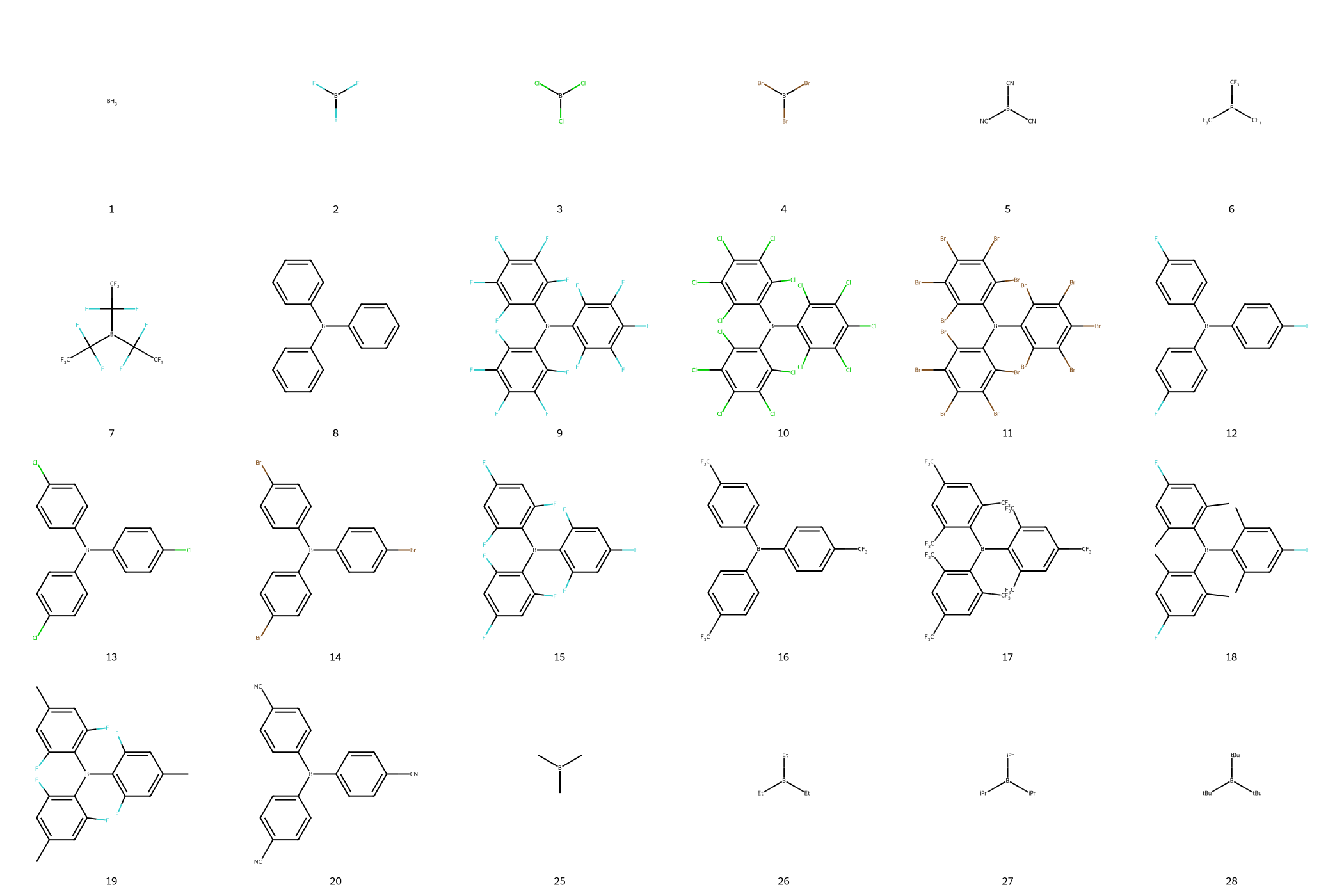

Supplement: SC-017-D5SC09530A-s001 [file SC-017-D5SC09530A-s001.zip › figures_si/acids.png]

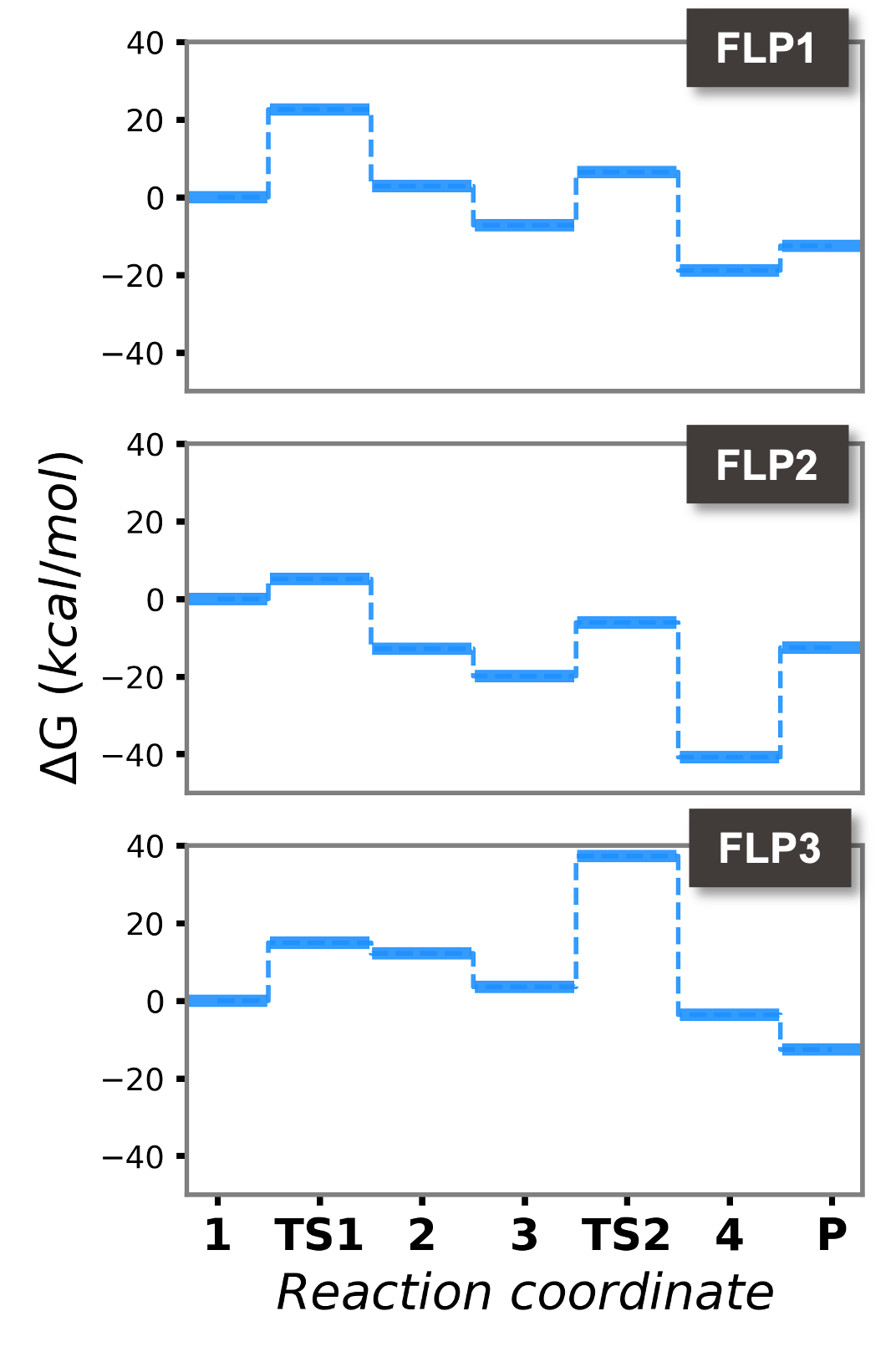

Supplement: SC-017-D5SC09530A-s001 [file SC-017-D5SC09530A-s001.zip › figures_si/additional-flp-profiles.png]

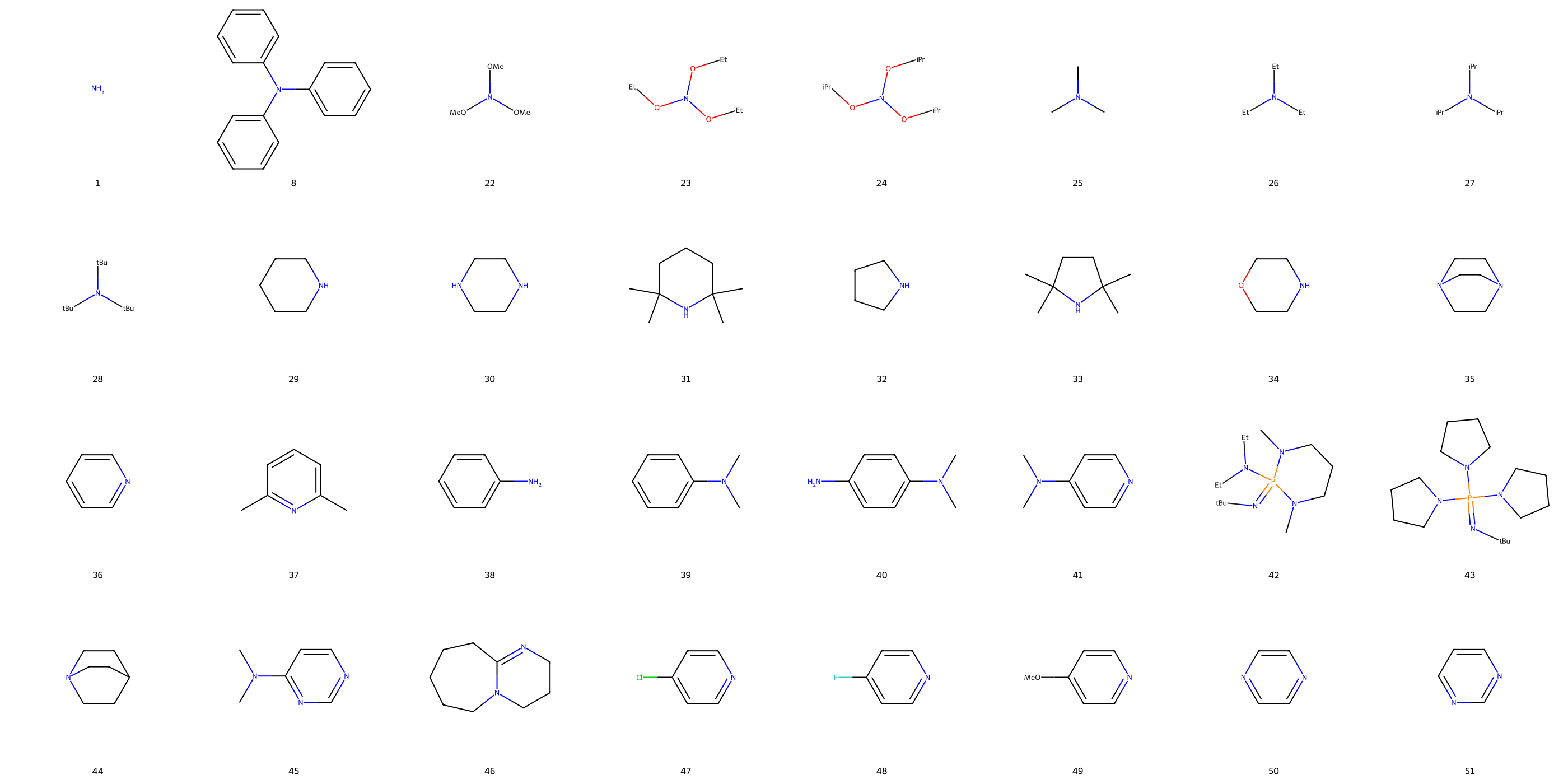

Supplement: SC-017-D5SC09530A-s001 [file SC-017-D5SC09530A-s001.zip › figures_si/bases.png]

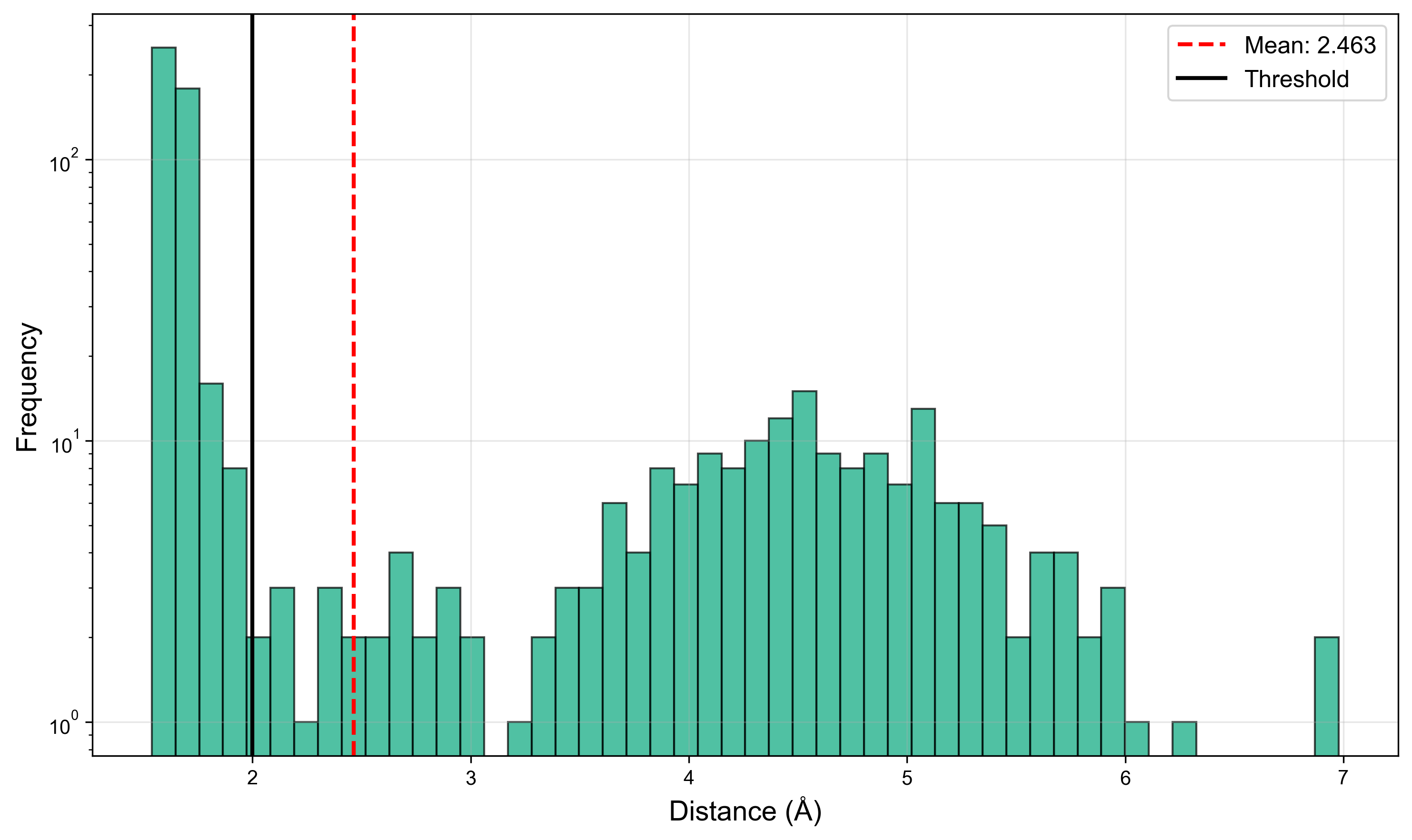

Supplement: SC-017-D5SC09530A-s001 [file SC-017-D5SC09530A-s001.zip › figures_si/bwdistance_histogram.png]

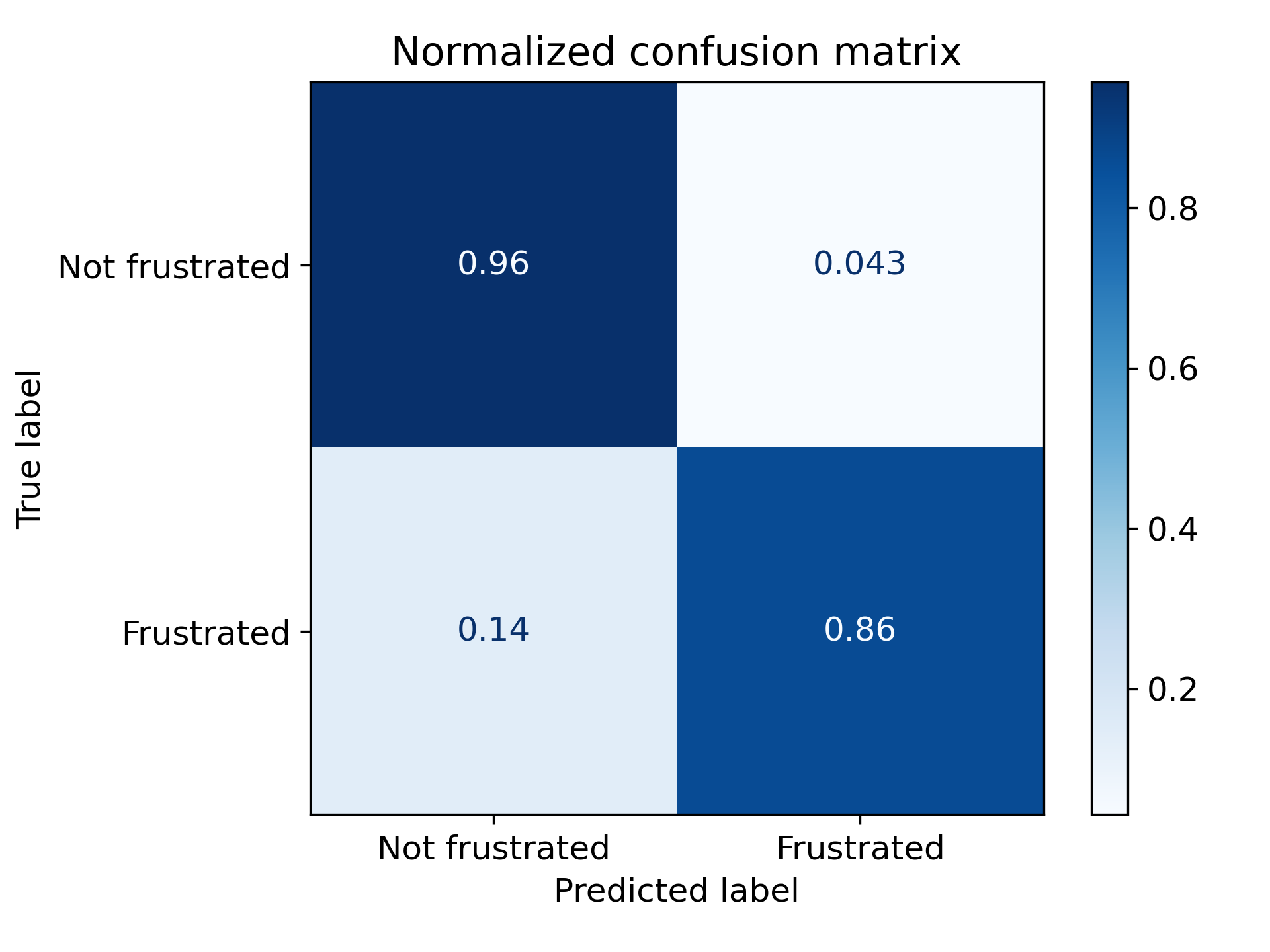

Supplement: SC-017-D5SC09530A-s001 [file SC-017-D5SC09530A-s001.zip › figures_si/confusion_matrix_normalized.png]

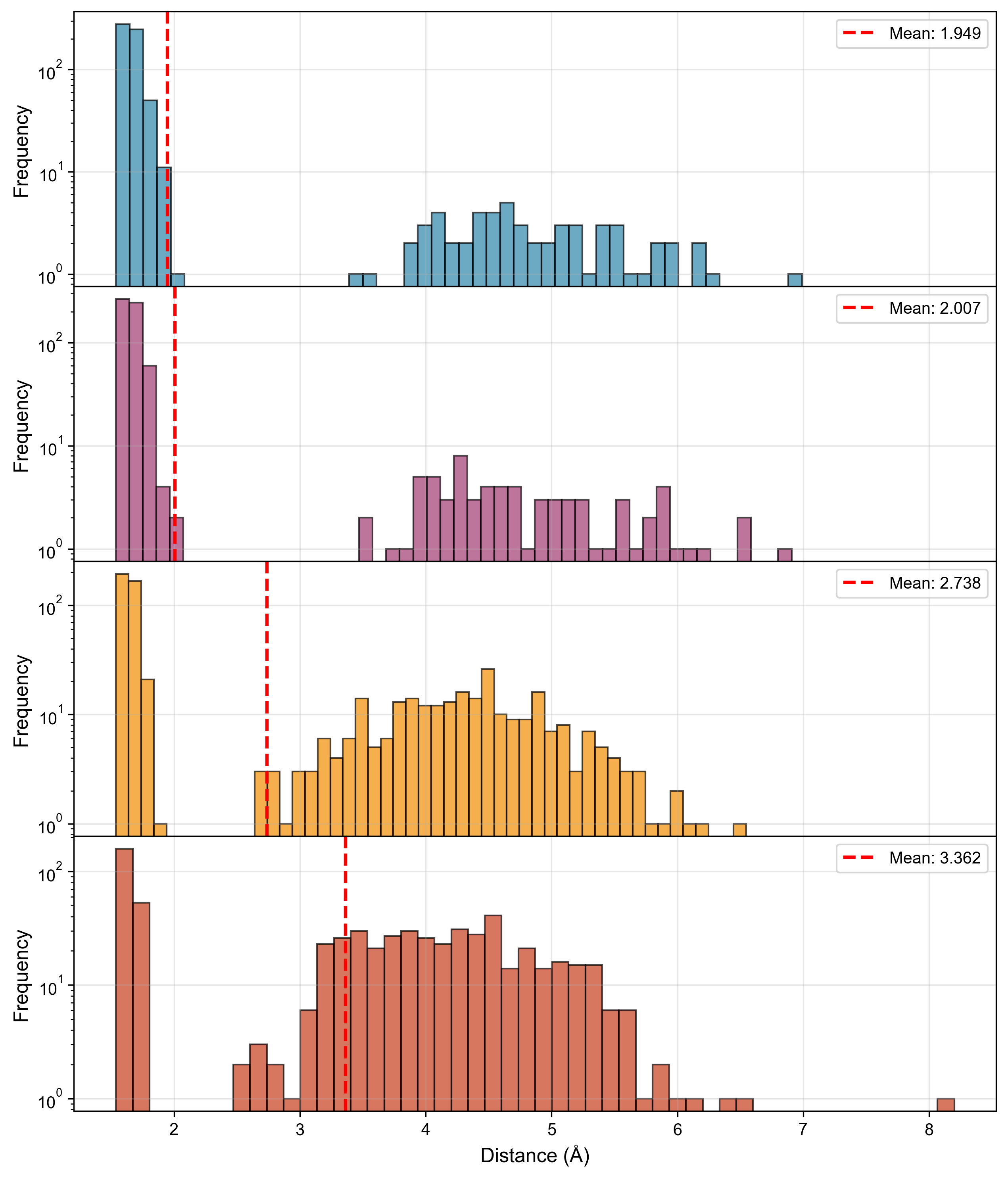

Supplement: SC-017-D5SC09530A-s001 [file SC-017-D5SC09530A-s001.zip › figures_si/distance_histograms_multipanel.png]

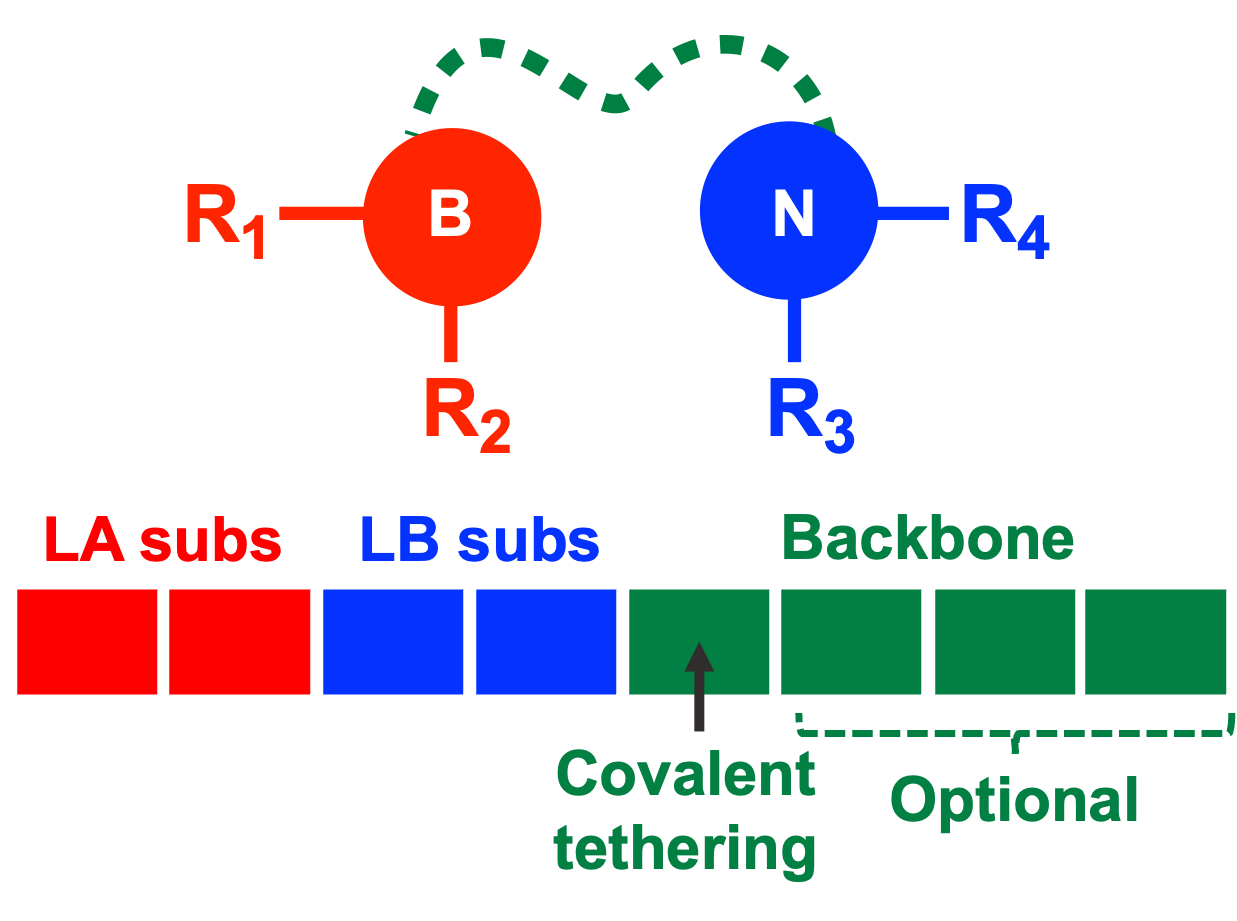

Supplement: SC-017-D5SC09530A-s001 [file SC-017-D5SC09530A-s001.zip › figures_si/flp-chromosome.png]

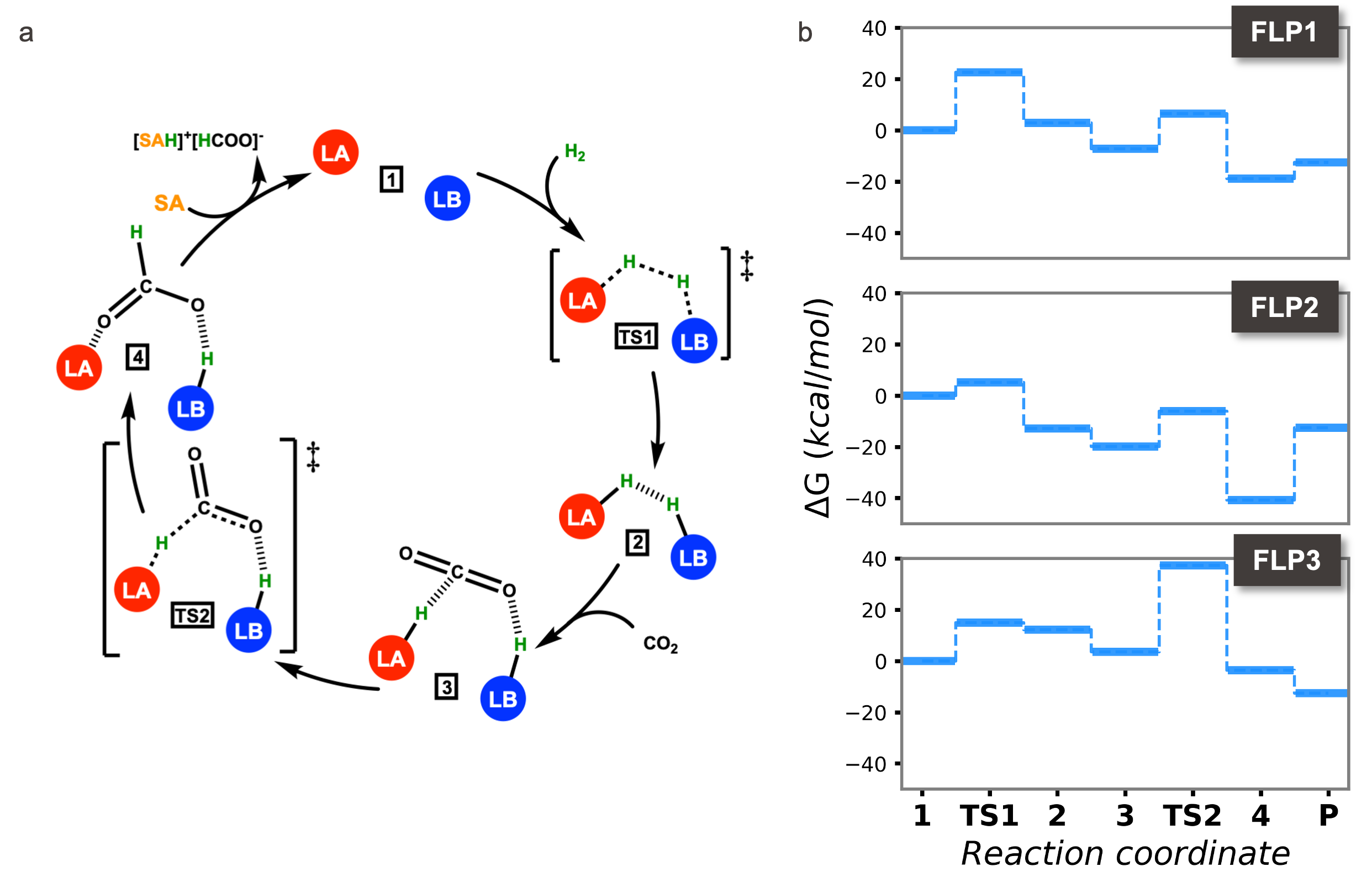

Supplement: SC-017-D5SC09530A-s001 [file SC-017-D5SC09530A-s001.zip › figures_si/flp123.png]

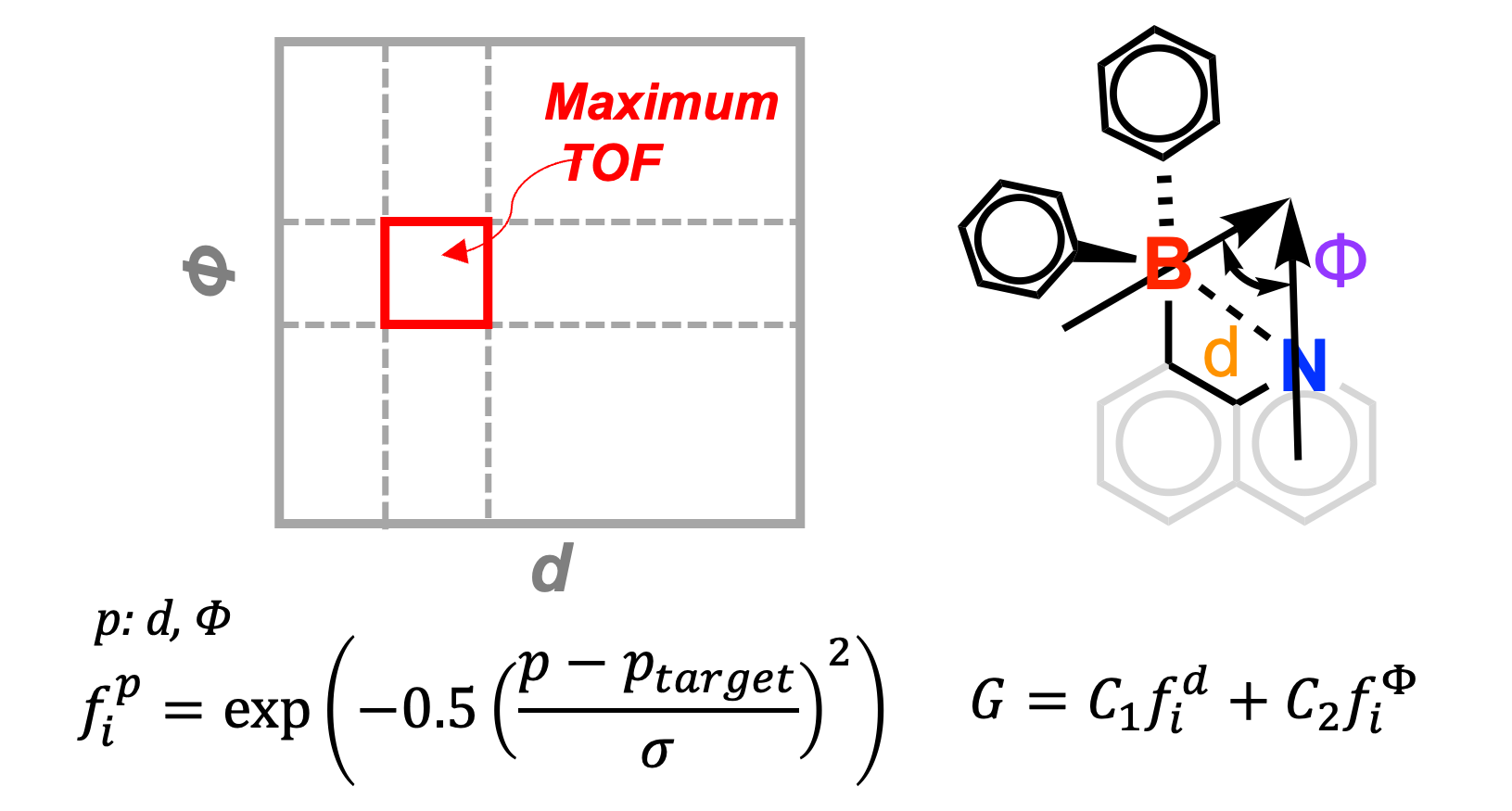

Supplement: SC-017-D5SC09530A-s001 [file SC-017-D5SC09530A-s001.zip › figures_si/geom-score.png]

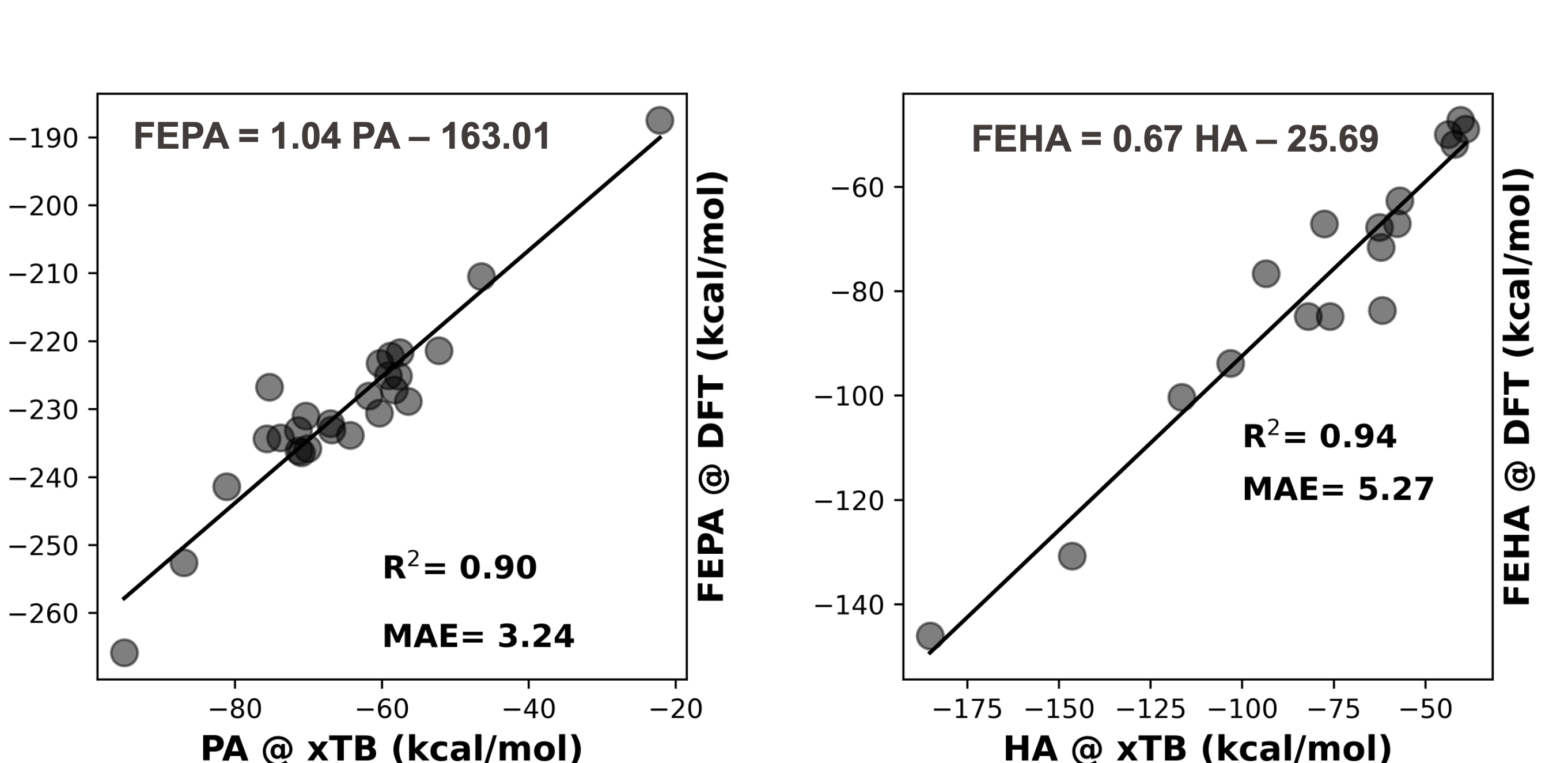

Supplement: SC-017-D5SC09530A-s001 [file SC-017-D5SC09530A-s001.zip › figures_si/pa-ha-corr.png]

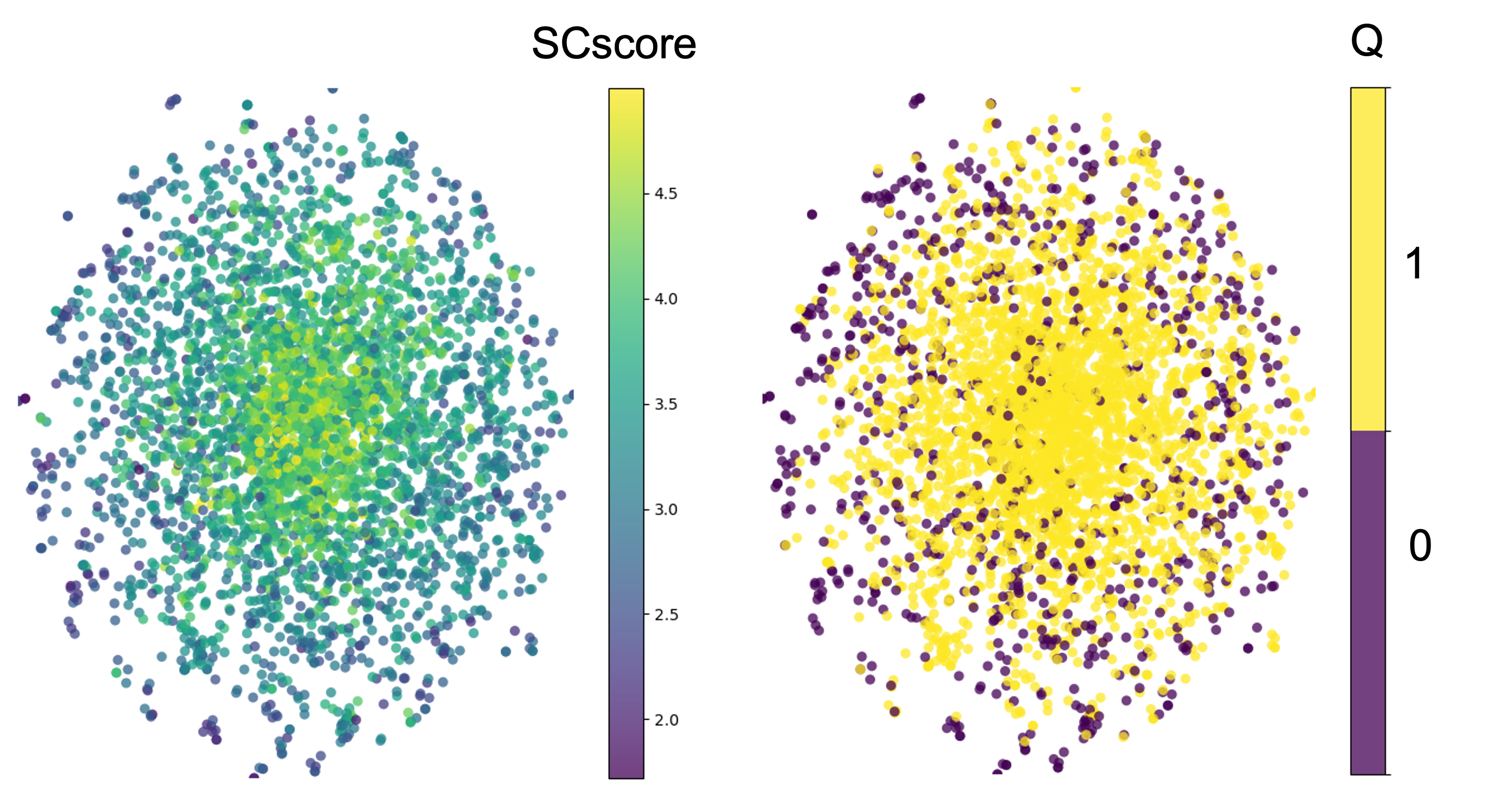

Supplement: SC-017-D5SC09530A-s001 [file SC-017-D5SC09530A-s001.zip › figures_si/scs_Q_tradeoff.png]
