# Supplementary material for: Inverse design of frustrated Lewis pairs for direct catalytic CO2 hydrogenation: refining and expanding design rules
Source: Chem Sci. 2026 Feb 5;17(14):7071–81. doi: 10.1039/d5sc09530a (PMC12903909; doi:10.1039/d5sc09530a)
Supplement: SC-017-D5SC09530A-s002 [file SC-017-D5SC09530A-s002.pdf]

Supplementary Information (SI) for Chemical Science.
This journal is © The Royal Society of Chemistry 2026

Electronic Supplementary Information (ESI) for Inverse Design of Frustrated Lewis Pairs for Direct Catalytic CO_2 Hydrogenation: Refining and Expanding Design Rules

Shubhajit Das,[†,‡] Ruben Laplaza,[†,¶] Thanapat Worakul,[†] and Clémence Corminboeuf[*,†,¶]

†*Laboratory for Computational Molecular Design, Institute of Chemical Sciences and Engineering, École Polytechnique Fédérale de Lausanne (EPFL), 1015 Lausanne, Switzerland*

‡*Present Address: Chair of Theoretical Chemistry, Faculty of Chemistry and Food chemistry, Technische Universität Dresden, Bergstraße 66c, 01069 Dresden, Germany*

¶*National Centre for Competence in Research–Catalysis (NCCR–Catalysis), École Polytechnique Fédérale de Lausanne, Lausanne, Switzerland*

E-mail: clemence.corminboeuf@epfl.ch

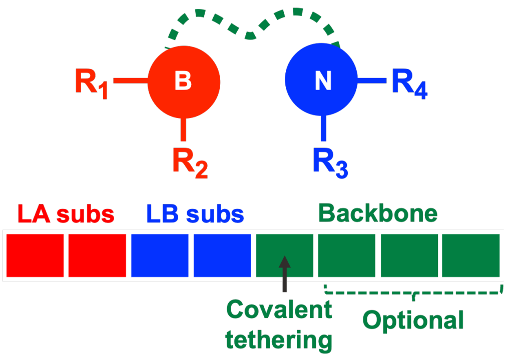

Scheme. S1. Schematic depiction of the FLP chrmosome composed of multiple genes corresponding to acid-base susbtituents and backbone.

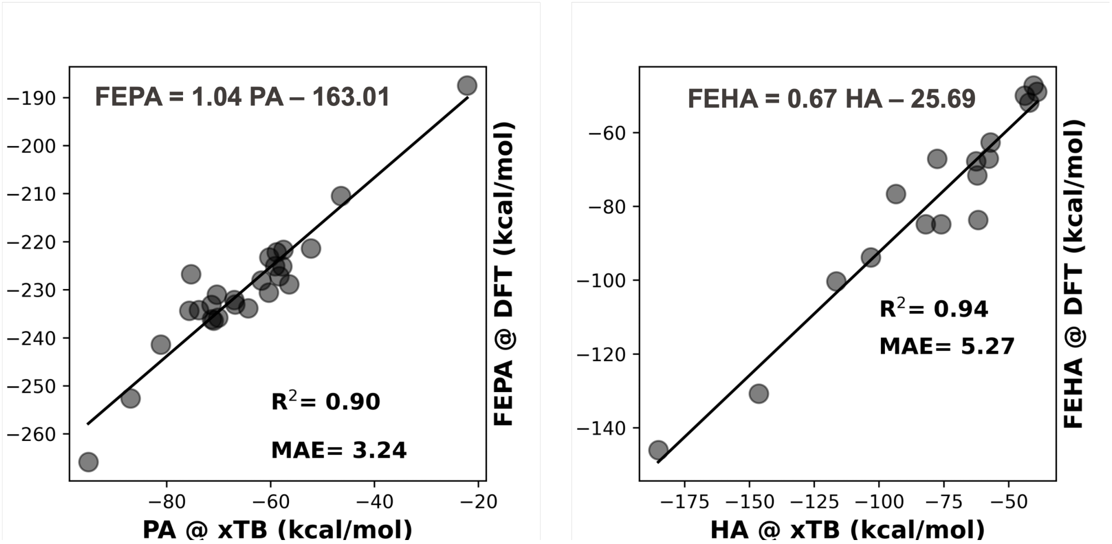

Fig. S1. Linear correlation between the GFN2-xTB computed proton affinity (right) and hydride affinity (left) values vs. the corresponding DFT computed FEPA and FEHA values, respectively.

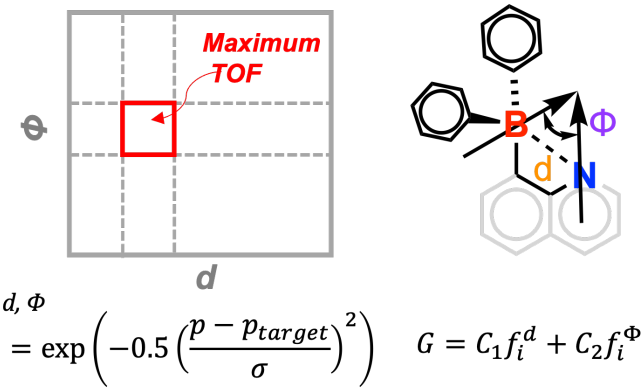

$$f_i^p = \exp\left(-0.5\left(\frac{p - p_{target}}{\sigma}\right)^2\right) \qquad G = C_1 f_i^d + C_2 f_i^\Phi$$

Fig. S2. Detailed descriptions of the angle and distance scores for evaluating the geometrical fitness of a given IFLP candidate.

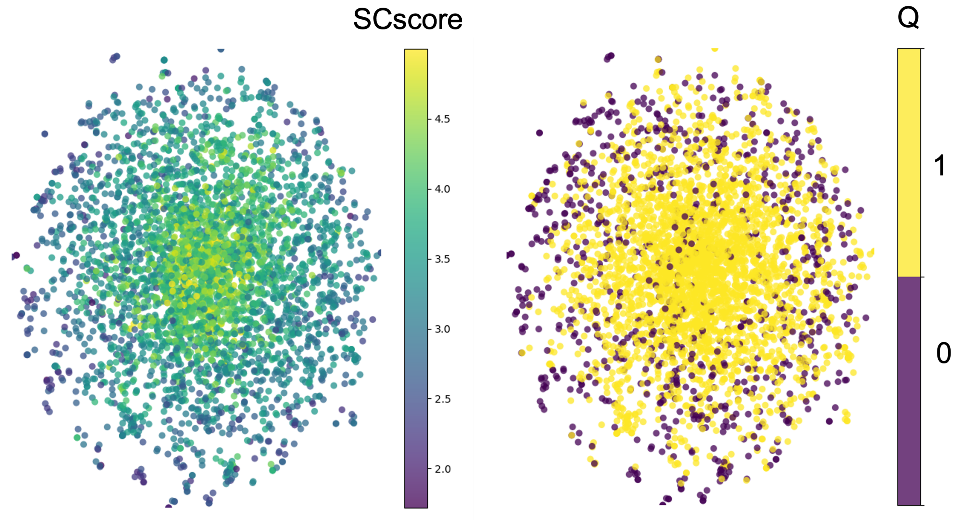

Fig. S3. 2D t-distributed stochastic neighbor embedding[1] (t-SNE) map of the random distribution of the IFLPs, where each IFLP is colored by the SCscore (left) and quenching score (right). The IFLPs were drawn randomly from the chemical space by performing a GA run with 100% mutation rate. The embedding was generated from the Morgan fingerprints of the IFLPs using RDkit.

S1: Computational details

Genetic optimization

All genetic optimization runs were performed using the NavicatGA software package[2] and the Chimera hierarchical scalarizer for multiobjective optimization.[3] The genetic optimization was performed using fixed 8-gene chromosomes to represent individual catalysts. Crossover

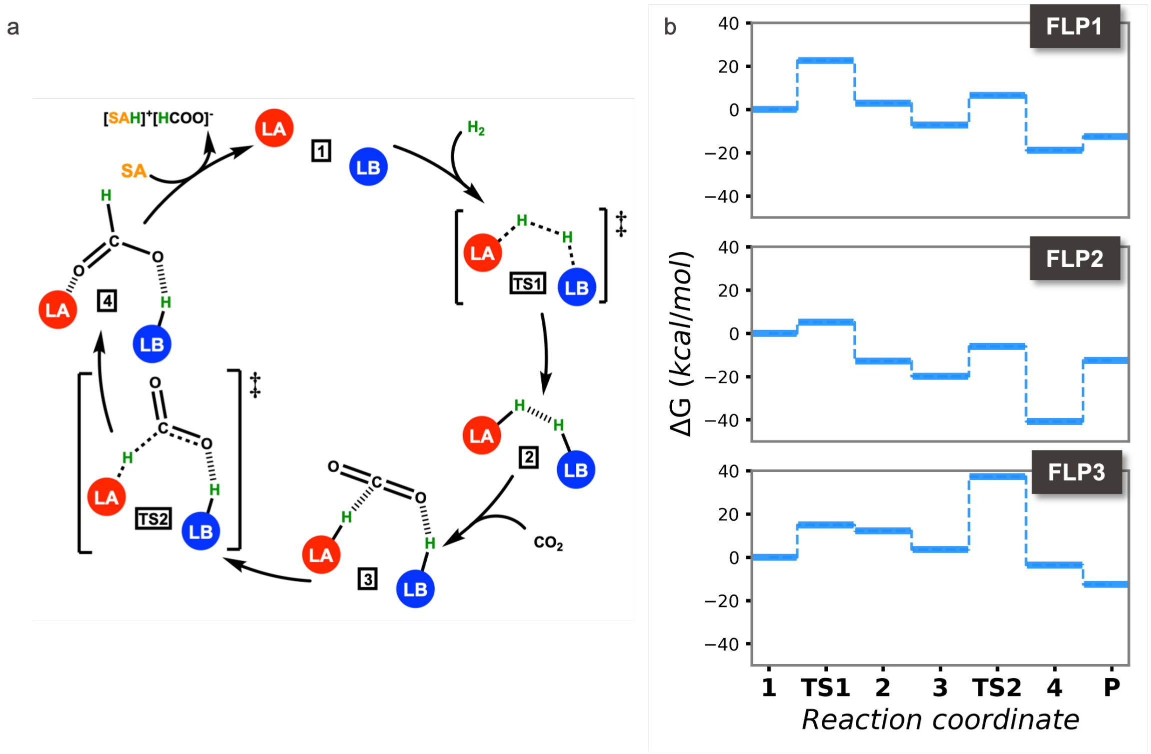

Fig. S4. (a) Catalytic cycle mechanism for FLP-catalyzed direct CTFH. (b) Computed CHTF profiles for **FLP1**, **FLP2**, and **FLP3**.

was performed by splicing the chromosomes with a single crossover point, and mutation is performed by randomly replacing genes by other valid genes (i.e., backbone genes can only replace backbone genes, etc.). The runs in the main text used the following parameters:

The code and data required to reproduce our results is available at `https://github.com/lcmd-epfl/ga_flp`. For more information on the general inner working of NavicatGA, we refer the reader to the original publication and documentation.[2]

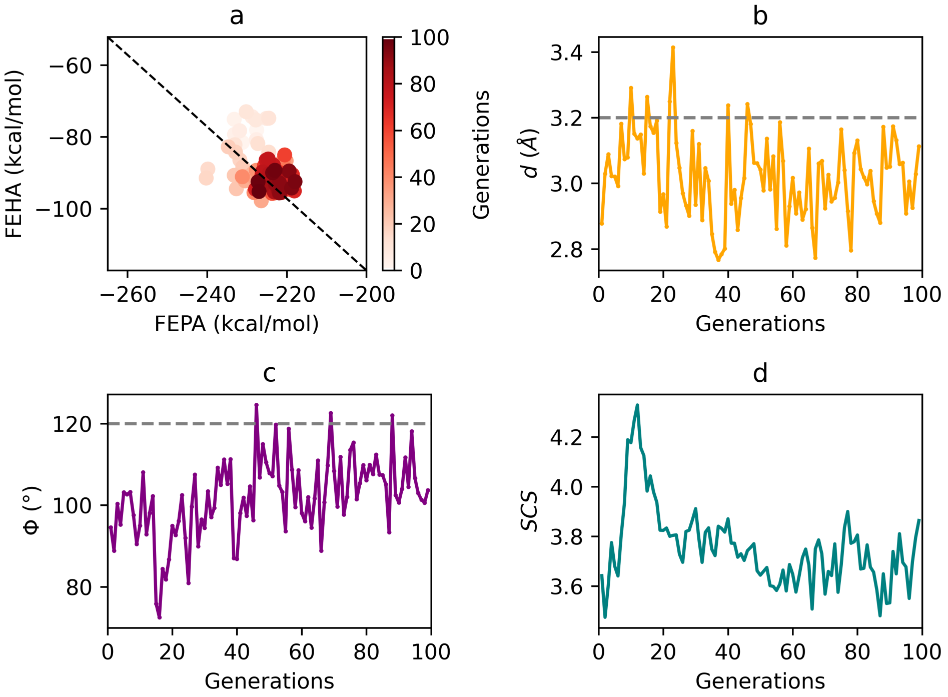

Fig. S5. Evolution of the chemical descriptors, FEPA and FEHA (a), geometric descriptors, d and Φ (b,c), and SCS for a multiobjective optimization run by an inverted objective hierarchy GS, CS followed by SCS. The average values of the properties over the population are shown. The black dotted line in the chemical descriptor space in (a) depicts the desired complementarity between FEPA and FEHA to achieve maximum TOF. The grey dashed lines in (b) and (c) indicate the target d and Φ values, respectively, used in the optimization. The top candidate extracted after 100 generations is shown in Figure 4f.

Computational details for the CHTF profiles of the lead FLPs

All DFT computations were performed at the PBE0[4,5]-D3(BJ)[6,7] level as implemented in the Gaussian 16 software.[8] For optimization of the molecular geometries of the reaction intermediates and transitional states, the def2-SVP basis set was used, while further single-point calculations were performed with a larger def2-TZVP basis.[9] The effect of entropic contributions, free energy corrections considering only vibrational contributions (corrected using the rigid-rotor approximation were included using the GoodVibes program[10]) were added to the single-point energies at the PBE0-D3(BJ)/def2-TZVP level of theory. All calculations were performed in the gas phase and the relative stabilities of the stationary points involved in the catalytic cycle were estimated as a difference in free

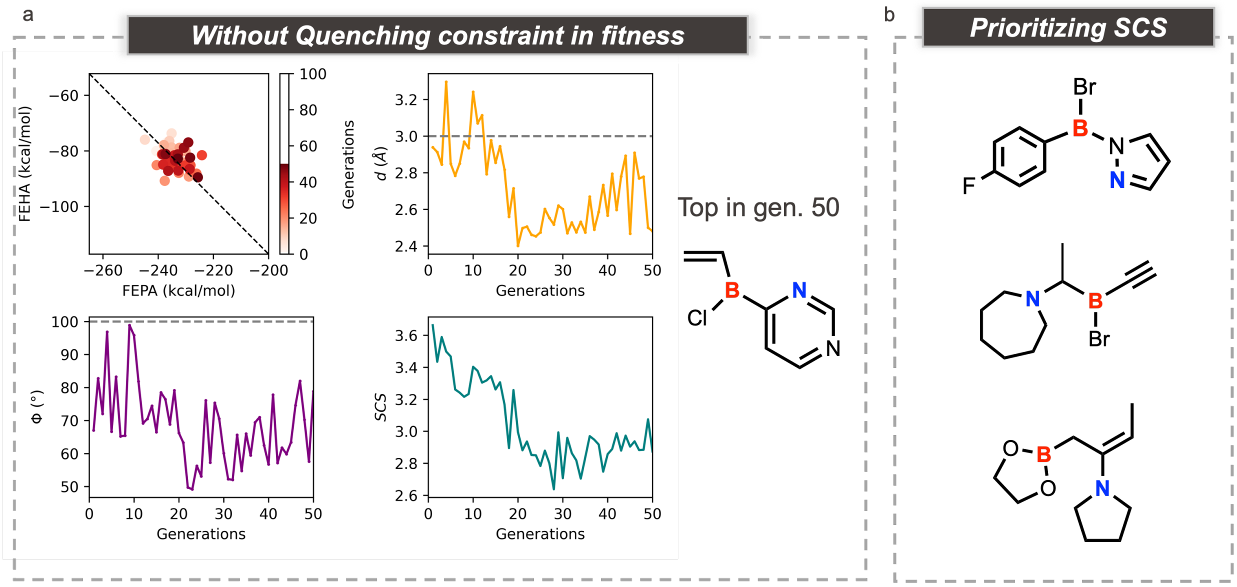

Fig. S6. (a) Evolution of the chemical descriptors, FEPA and FEHA (a), geometric descriptors, d and Φ, and SCS for a multiobjective optimization run by an objective hierarchy SCS and CS followed by GS, CS. The average values of the properties over the population are shown. The black dotted line in the chemical descriptor space in (a) depicts the desired complementarity between FEPA and FEHA to achieve maximum TOF. The grey dashed lines in distance and angle descriptor space indicate the target d and Φ values, respectively, used in the optimization. The top candidate extracted after 50 generations is shown on the right. (b) Top candidates obtained from optimization runs with objective hierarchy SCS, CS followed by GS.

energies. To maintain consistency with our previous works and to obtain a direct comparison, we used a pyridine LB molecule as the sacrificial agent.[11,12]

S2: Dimer Quenching Classifier

To predict the likelihood of intramolecular quenching in frustrated Lewis pairs (FLPs), we constructed a dataset and used it to train a random forest classifier. Dataset generation began with a collection of (intermolecular) FLP candidates, combining 32 amines and 24 boranes (Figs. S6 and S7). For each FLP, four conformers were generated by performing geometry optimizations at the GFN2-xTB level using xtb,[13] where the initial B···N distance was set at 3, 4, 5, and 6 Å, respectively. 647 pairs finished all optimization runs without error,

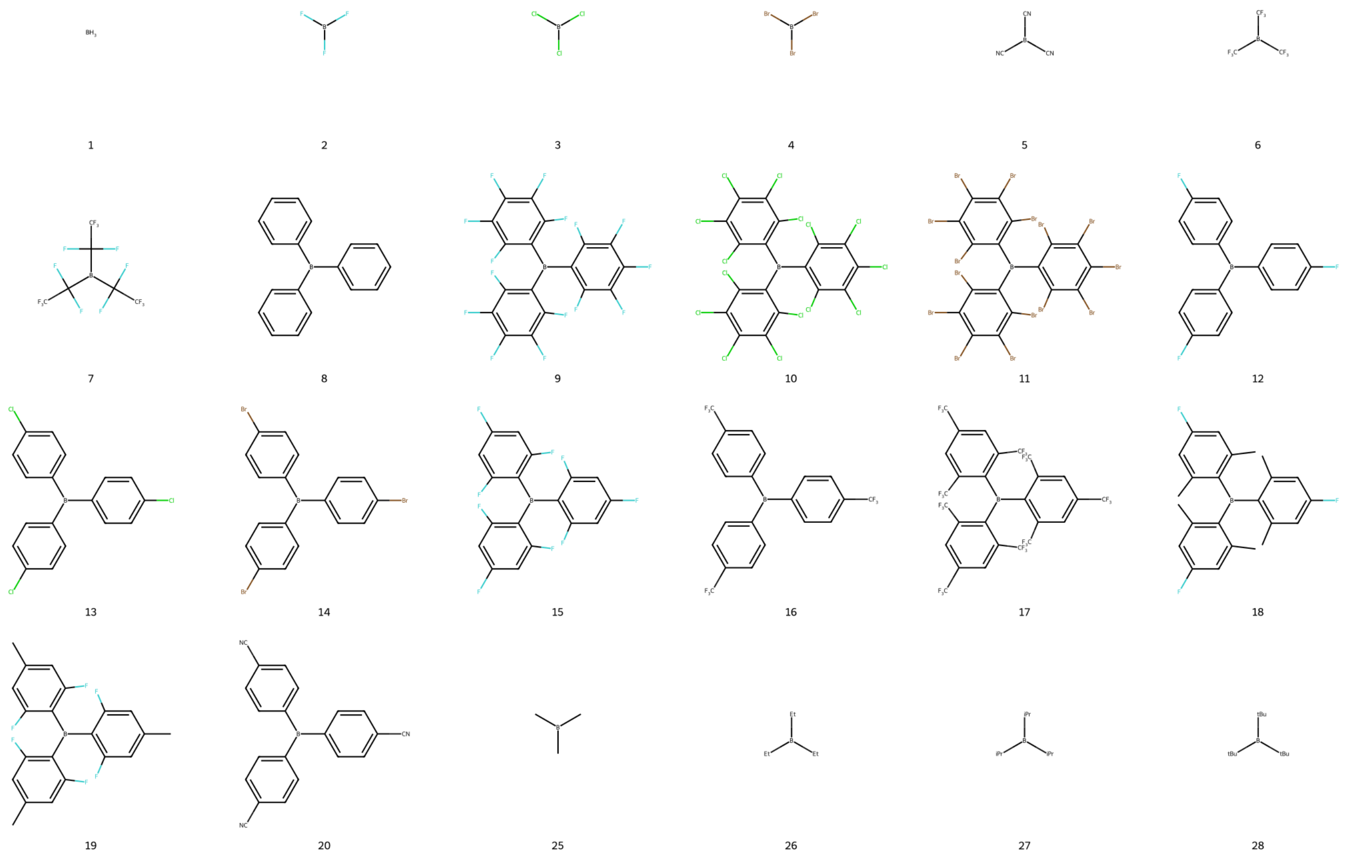

Fig. S7. 2D depiction of the Lewis acids used to generate the training data for the dimer quenching classifier. Numbering corresponds to the one used in the database.

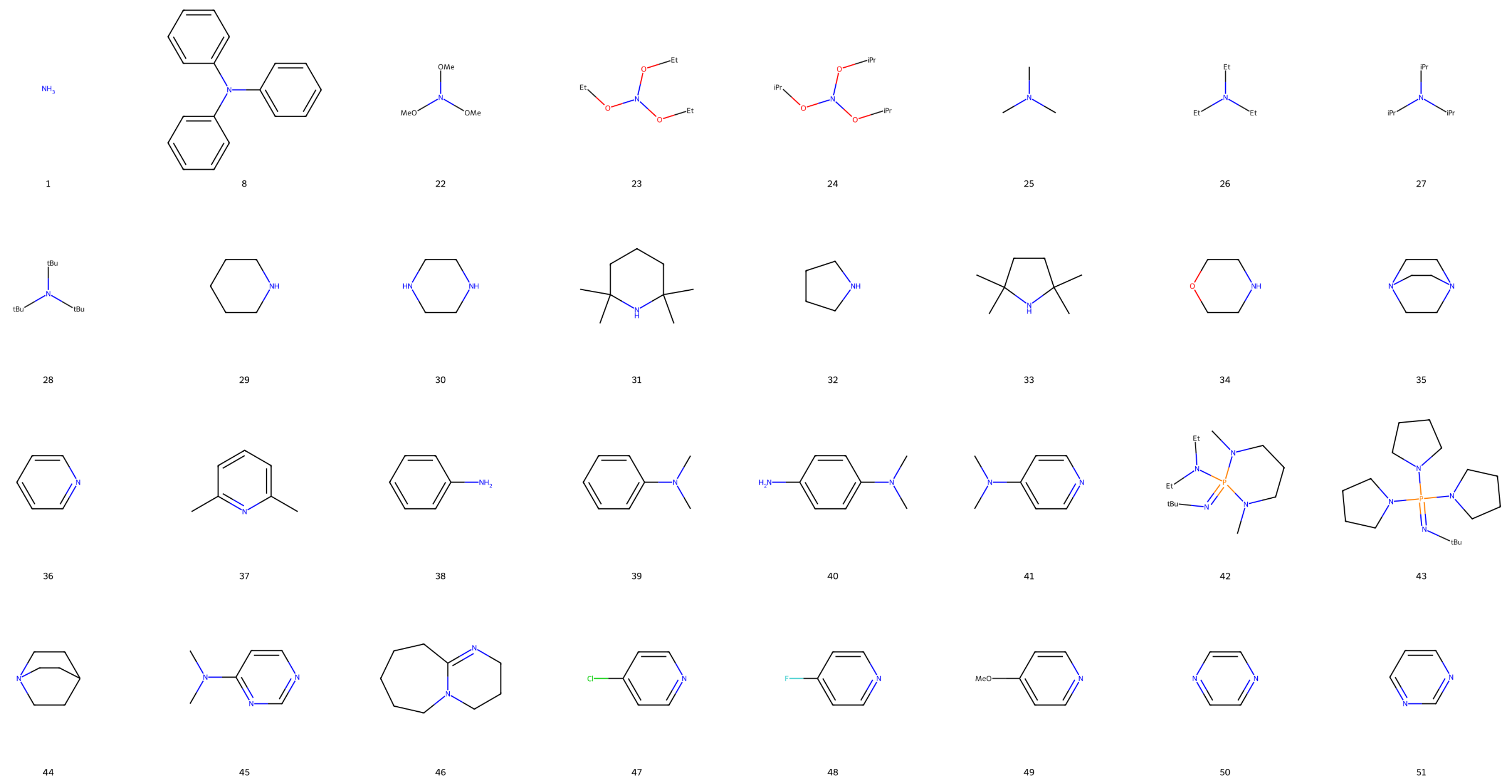

Fig. S8. 2D depiction of the Lewis bases used to generate the training data for the dimer quenching classifier. Numbering corresponds to the one used in the database.

Table S1. Parameters used in the genetic optimization runs.

ID	N_{genes}	P_{size}	M_{rate}	N_{cpts}	N_{cycles}	rtol1	rol2	rol3
R1	8	20	0.25	1	10	0.25	0.10	0.25
R2	8	20	0.25	1	100	0.10	0.25	0.25
R3	8	50	0.25	1	100	0.10	0.25	0.25
R4	8	20	0.25	1	50	0.25	0.10	0.25
R5	8	20	0.25	1	50	0.25	0.10	0.25
R6	8	20	0.25	1	50	0.25	0.10	0.25
R7	8	20	0.25	1	50	0.25	0.10	0.25
R8	8	20	0.25	1	50	0.25	0.10	0.25
R9	8	20	0.25	1	50	0.25	0.10	0.25
R9	8	20	0.25	1	50	0.25	0.10	0.25
R10	8	50	0.25	1	100	0.25	0.10	0.25

Default GA parameters (unless stated otherwise): N_{genes}=`8`, P_{size}=`20`, M_{rate}=`0.25`, N_{cpts}=`1`, N_{cycles}=`50`. Tolerances are the Chimera scalarizer settings `(t1, t2, t3)` in hierarchical order. The run IDs follow the numbering of the lead FLP candidates mentioned in the main manuscript.

out of the total 768 pairs tested. From each optimized structure, the single-point energy and the final, optimized B···N distance between the Lewis acid and the Lewis base were extracted (Fig. S8). These values were assembled into a single table containing one row per FLP with four corresponding energy–distance pairs. The range of initial distances was meant to allow the acid and pair to proceed to either a fully quenched adduct or not, depending on the frustrating effect of the acid and base substituents. Some combinations formed dative adducts under all circumstances due to the lack of any steric frustration, whereas others were always frustrated and promptly repelled each other when the optimization runs started from short distances. A few cases were in-between, and collapsed to the dative adduct only if the initial geometry had a short distance.

To account for these intermediate cases, the target label (quenched vs. non-quenched) was assigned using a Boltzmann-weighted average B···N distance. Relative conformer energies (ΔE) were computed across all four final optimized geometries, and Boltzmann weights (at 298.15 K) were evaluated from these energies. The Boltzmann-weighted distance

(BWDistance) was then obtained as the weighted average of the four B···N distances. Note that this approach, based on simple geometry relaxation, does not capture the kinetics of frustration. We deliberately chose to neglect the possibility of an energy barrier associated with the transition between the dative adduct and the frustrated non-covalently bound configuration due to the inherent complexity of studying such a process. Nonetheless, we reasoned that our main concern is identifying (and discarding) LA/LB combinations where adduct formation is favored thermodynamically. In doing so, we accept that there could be a small number of cases where we are being overly conservative and kinetics could contribute to the frustration even if the process is thermodynamically favored.

We observed that Lewis acid and base pairs that are amenable to quenching and dative adduct formation have a strong energy preference for short (bonding) B···N distances, resulting in a BWDistance under 2.0 Å, while FLPs are more stable at greater distances instead due to steric repulsion, leading to a larger BWDistance. FLPs with BWDistance greater than 2.0 Å were labeled as non-quenched (frustrated, label = 1), indicating persistent separation of the donor–acceptor pair, whereas those below this threshold were labeled as quenched (label = 0), corresponding to situations in which collapse into a classical adduct is both likely and energetically favourable (see Fig. S9). Note that the BWDistance distribution is approximately bimodal with the 2 Å threshold being near the separation point. Only 20 entries out of 647 (3%) are near the decision threshold.

To quantify steric effects, we calculated buried-volume descriptors around the Lewis acid and base centers. For each FLP, the acid and base fragments were isolated, and the Morfeus[14] library was used to compute the percent buried volume ($\%V_{bur}$) around the boron (acid) and nitrogen (base) atoms of the isolated components. These calculations were performed using probe radii of 2.0, 2.5, 3.0, and 3.5 Å, capturing steric encumbrance at multiple length scales and yielding eight steric descriptors per FLP. These eight buried-volume descriptors (four from the Lewis acid and four from the Lewis base) are the input of the dimer quenching classifier.

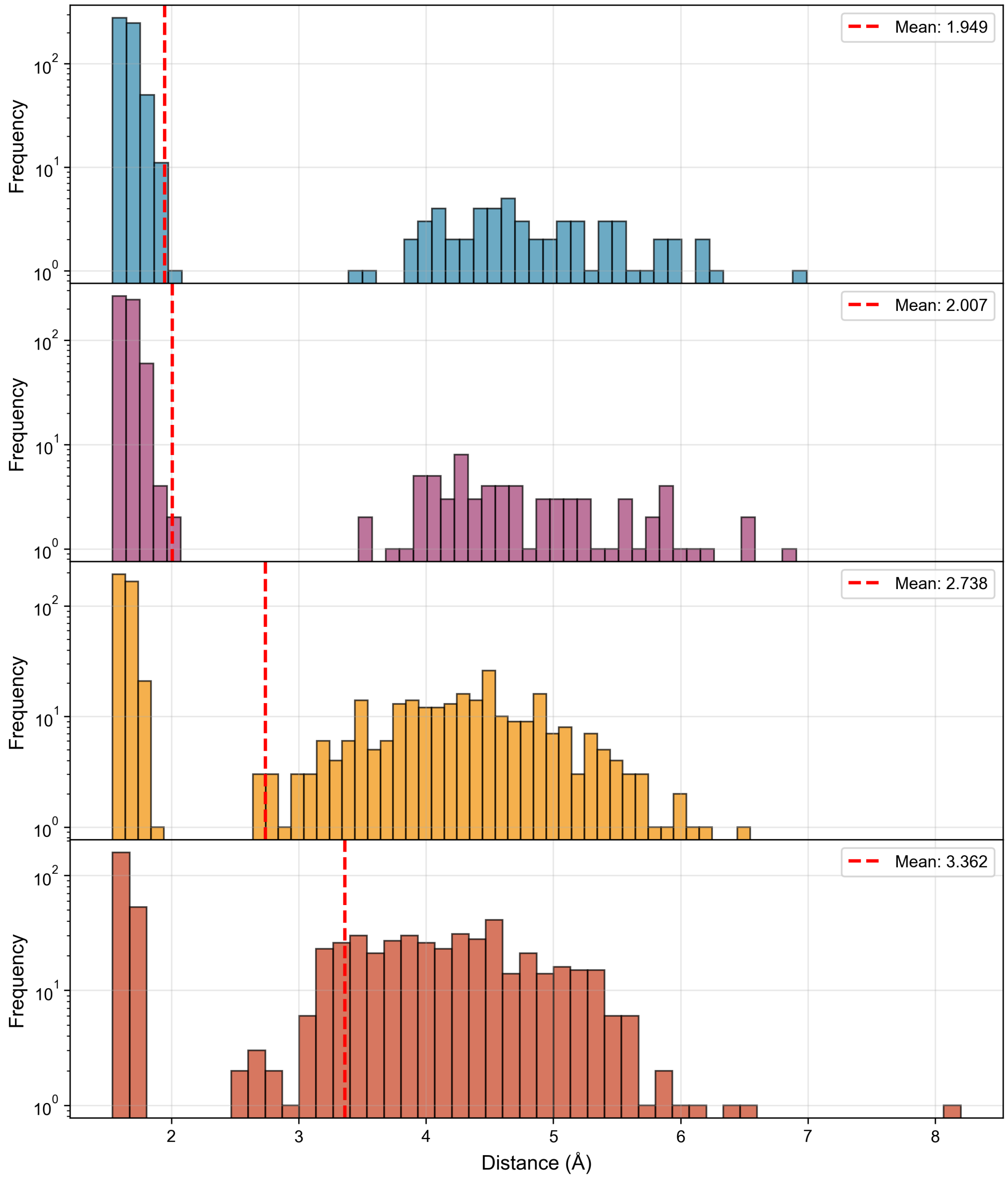

Fig. S9. Histogram of the optimized B···N distances of the 647 Lewis acid and base pairs used to train the dimer quenching classifier. Subplots correspond to the final optimized distances starting from a B···N distance of 3, 4, 5, and 6 Å, respectively.

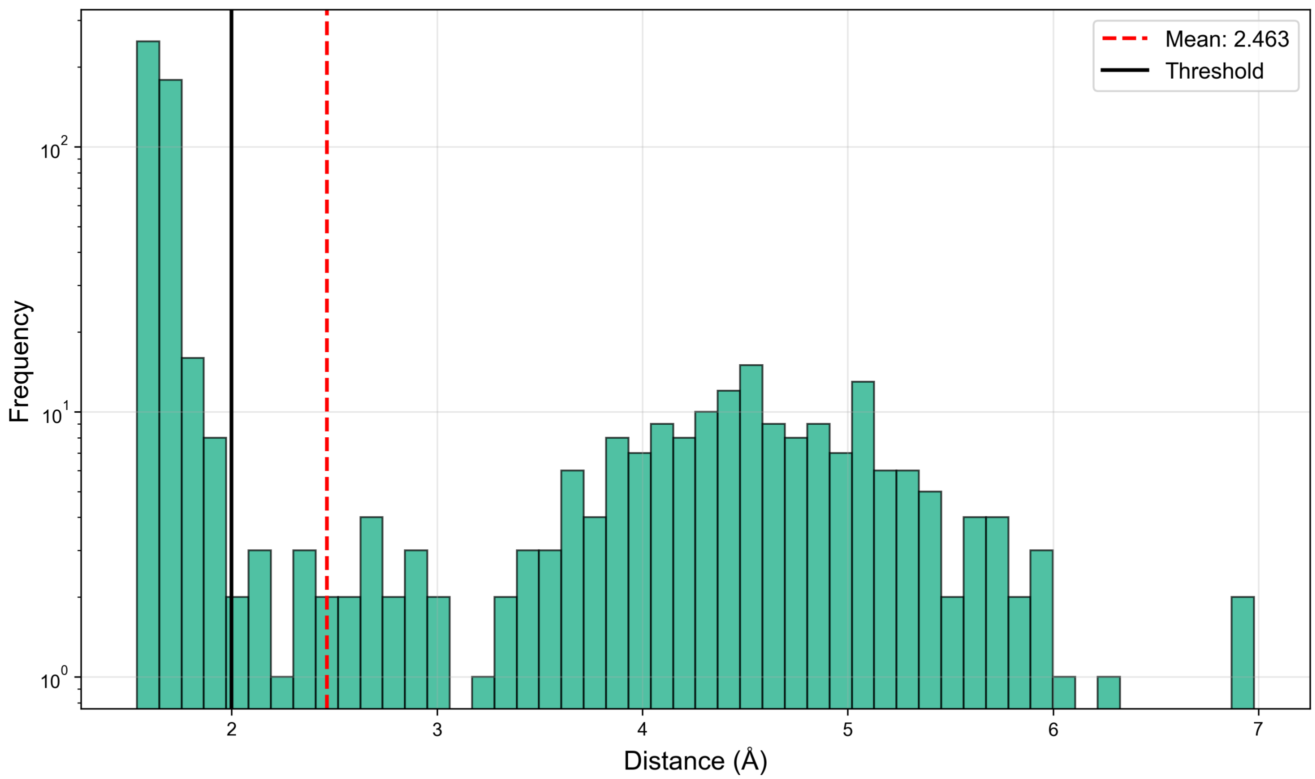

Fig. S10. Histogram of the Boltzmann-weighted distance of the 647 Lewis acid and base pairs used to train the dimer quenching classifier. The entries correspond to the Boltzmann-weighted average of Fig. S0. The black vertical line highlights the 2.0 Å threshold for frustration.

The final dataset combined these eight buried-volume features with the binary frustration label. The curated dataset, along with the geometries of these molecules, are available publicly at `https://github.com/lcmd-epfl/ga_flp/tree/main/data/quenching`. We then trained a scikit-learn[15] random forest classifier using the eight buried-volume features to predict the frustration label. We optimized the model through a grid search over several hyperparameters, including the number of estimators in the forest (50, 100, 200, 500), the number of features to consider when looking for the best split ('sqrt', 'log2'), and whether bootstrap samples are used when building trees. The best-performing model has a number of estimators of 500, with the number of features to consider being log2 and bootstrap set to False. We noticed that the classifier was remarkably accurate, as shown in the confusion matrix in Fig. S11. In particular, we highlight that the model very rarely

predicts a frustrated label in not frustrated pairs, meaning that the inclusion of the dimer quenching classifier in our fitness function is unlikely to (falsely) accept invalid candidates. The script to train or predict with our dimer quenching model architecture is available at `https://github.com/lcmd-epfl/ga_flp/blob/main/ga_core/frustration_c.py`.

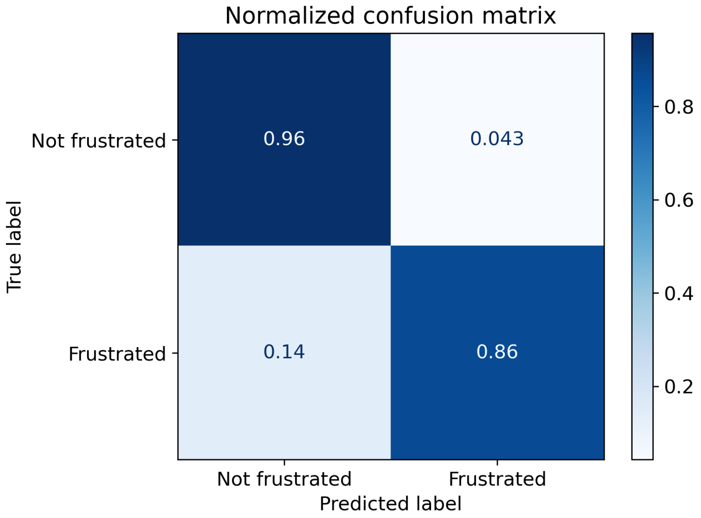

Fig. S11. Normalized confusion matrix from the trained random forest classifier. Predictions are aggregated from 20 test folds in a 20-fold splitting loop.

Table S2: Synthetic complexity score (SCS) of the top candidates FLP1-FLP10.

	SCScore
FLP1	2.83
FLP2	3.54
FLP3	3.59
FLP4	2.29
FLP5	3.40
FLP6	4.18
FLP7	3.91
FLP8	4.20
FLP9	3.65
FLP10	3.44

References

(1) Van der Maaten, L.; Hinton, G. Visualizing data using t-SNE. *J. Mach. Learn. Res.* **2008**, *9*, 2579–2605.

(2) Laplaza, R.; Gallarati, S.; Corminboeuf, C. Genetic Optimization of Homogeneous Catalysts. *Chemistry–Methods* **2022**, *2*, e202100107.

(3) Häse, F.; Roch, L. M.; Aspuru-Guzik, A. Chimera: enabling hierarchy based multi-objective optimization for self-driving laboratories. *Chem. Sci.* **2018**, *9*, 7642–7655.

(4) Perdew, J. P.; Burke, K.; Ernzerhof, M. Generalized gradient approximation made simple. *Phys. Rev. Lett.* **1996**, *77*, 3865.

(5) Adamo, C.; Barone, V. Toward reliable density functional methods without adjustable parameters: The PBE0 model. *J. Chem. Phys.* **1999**, *110*, 6158–6170.

(6) Grimme, S.; Antony, J.; Ehrlich, S.; Krieg, H. A consistent and accurate ab initio parametrization of density functional dispersion correction (DFT-D) for the 94 elements H-Pu. *J. Chem. Phys.* **2010**, *132*, 154104.

(7) Grimme, S.; Ehrlich, S.; Goerigk, L. Effect of the damping function in dispersion corrected density functional theory. *J. Comput. Chem.* **2011**, *32*, 1456–1465.

(8) Frisch, M. J.; Trucks, G. W.; Schlegel, H. B.; Scuseria, G. E.; Robb, M. A.; Cheeseman, J. R.; Scalmani, G.; Barone, V.; Petersson, G. A.; Nakatsuji, H. et al. Gaussian 16 Revision A.03. 2016; Gaussian Inc. Wallingford CT.

(9) Weigend, F.; Ahlrichs, R. Balanced basis sets of split valence, triple zeta valence and quadruple zeta valence quality for H to Rn: Design and assessment of accuracy. *Phys. Chem. Chem. Phys.* **2005**, *7*, 3297–3305.

(10) Funes-Ardoiz, I.; Paton, R. GoodVibes v2. 0.2. *DOI:http://doi.org/10.5281/zenodo.595246* **2019**, 291.

(11) Das, S.; Laplaza, R.; Blaskovits, J. T.; Corminboeuf, C. Mapping Active Site Geometry to Activity in Immobilized Frustrated Lewis Pair Catalysts. *Angew. Chem. Int. Ed.* **2022**, *61*, e202202727.

(12) Das, S.; Laplaza, R.; Blaskovits, J. T.; Corminboeuf, C. Engineering Frustrated Lewis Pair Active Sites in Porous Organic Scaffolds for Catalytic CO2 Hydrogenation. *J. Am. Chem. Soc.* **2024**, *146*, 15806–15814.

(13) Bannwarth, C.; Ehlert, S.; Grimme, S. GFN2-xTB—An accurate and broadly parametrized self-consistent tight-binding quantum chemical method with multipole electrostatics and density-dependent dispersion contributions. *J. Chem. Theory. Comput.* **2019**, *15*, 1652–1671.

(14) Jorner, K.; Turcani, L. kjelljorner/morfeus: v0.7.2. 2022; `https://doi.org/10.5281/zenodo.7017599`.

(15) Pedregosa, F.; Varoquaux, G.; Gramfort, A.; Michel, V.; Thirion, B.; Grisel, O.; Blondel, M.; Prettenhofer, P.; Weiss, R.; Dubourg, V. et al. Scikit-learn: Machine Learning in Python. *J. Mach. Learn. Res.* **2011**, *12*, 2825–2830.
